# Very Small Embryonic-Like Stem Cells Purified from Umbilical Cord Blood Lack Stem Cell Characteristics

**DOI:** 10.1371/journal.pone.0034899

**Published:** 2012-04-03

**Authors:** Ralitza Danova-Alt, Andreas Heider, Dietmar Egger, Michael Cross, Rüdiger Alt

**Affiliations:** 1 Vita 34 AG, Leipzig, Germany; 2 Translational Centre for Regenerative Medicine (TRM), Universität Leipzig, Leipzig, Germany; 3 Department of Hematology, Oncology and Hemostaseology, Universität Leipzig, Leipzig, Germany; French Blood Institute, France

## Abstract

Very small embryonic-like (VSEL) cells have been described as putatively pluripotent stem cells present in murine bone marrow and human umbilical cord blood (hUCB) and as such are of high potential interest for regenerative medicine. However, there remain some questions concerning the precise identity and properties of VSEL cells, particularly those derived from hUCB. For this reason, we have carried out an extensive characterisation of purified populations of VSEL cells from a large number of UCB samples. Consistent with a previous report, we find that VSEL cells are CXCR4^+^, have a high density, are indeed significantly smaller than HSC and have an extremely high nuclear/cytoplasmic ratio. Their nucleoplasm is unstructured and stains strongly with Hoechst 33342. A comprehensive FACS screen for surface markers characteristic of embryonic, mesenchymal, neuronal or hematopoietic stem cells revealed negligible expression on VSEL cells. These cells failed to expand in vitro under a wide range of culture conditions known to support embryonic or adult stem cell types and a microarray analysis revealed the transcriptional profile of VSEL cells to be clearly distinct both from well-defined populations of pluripotent and adult stem cells and from the mature hematopoietic lineages. Finally, we detected an aneuploid karyotype in the majority of purified VSEL cells by fluorescence in situ hybridisation. These data support neither an embryonic nor an adult stem cell like phenotype, suggesting rather that hUCB VSEL cells are an aberrant and inactive population that is not comparable to murine VSEL cells.

## Introduction

Over the last ten years, there have been various reports of the isolation of putatively pluripotent cells from bone marrow, umbilical cord blood and other tissues [1]–[4]. However, many of these findings still await independent confirmation and there is increasing scepticism towards the existence of pluripotent stem cells in the adult [5]–[7]. Since the majority of the isolation procedures involve the in vitro culture of primary cells, there is always the possibility that culture variables make a decisive contribution to the emergence of pluripotency and that these have not been reproduced exactly between different laboratories.

The discovery of very small embryonic-like (VSEL) stem cells in adult bone marrow and umbilical cord blood promised to shed light on this controversy, since here for the first time was a highly versatile and putatively pluripotent cell type that could be isolated directly from primary tissue and investigated without the need for intervening culture [8], [9]. Murine VSEL cells have been described as a rare population of small cells which over express pluripotency genes including Oct4, Nanog and SOX2 at both the mRNA and protein levels, and maintain a demethylated Oct4 promotor. They have euchromatic nuclei and are reportedly able to adopt the phenotype of tissues from all three germ layers upon differentiation in vitro. However, unlike pluripotent ES, EC or iPS cells, murine bone marrow derived VSEL cells do not form teratomas in vivo [9], [10]. Furthermore no contribution of these cells to chimeric embryos has been demonstrated, so that evidence for classical pluripotency is still lacking. Thus, although it has been suggested that VSEL cells could play a role in regeneration and tissue repair, both their true physiological function and their origin remain uncertain.

Even less information is available concerning human VSEL cells, possibly because of reported difficulties with their isolation. They were initially described as a rare population of small cells within the non-hematopoietic CD45^−^Lin^−^ UCB population which should also contain the endothelial and mesenchymal progenitors frequently isolated from UCB [11]–[14]. In common with their murine counterparts, VSEL cells from hUCB reportedly have a high nucleus to cytoplasm ratio, euchromatic chromatin, and a high expression of pluripotency genes at mRNA and protein levels [8]. Furthermore, hVSEL cells have subsequently been reported to express the surface markers CXCR4, CD34 and CD133 characteristic of adult stem and progenitor populations, as well as the SSEA-4 antigen found on embryonic stem cells.

However, the representation of these markers and the relationship between them on the CD45^−^Lin^−^ population has not been investigated systematically so that the precise phenotype of human VSEL cells in this respect remains unclear. As an example, the initial description of human UCB-derived VSEL cells presented detailed immunophenotypic and electron microscopic analysis of CD45^−^Lin^−^CXCR4^+^ VSEL cells, while a later publication of the same group reports an isolation protocol based on magnetic enrichment of CD133^+^ cells, irrespective of any CXCR4 expression [8], [15]. As far as we are aware, these populations have not yet been characterized or compared functionally. In order to clarify the features of the hVSEL cells in more detail, we have established a comprehensive phenotypic profile of the Lin-CD45- population and optimised an isolation protocol for the Lin-CD45-CXCR4+ VSEL cells. Furthermore, we have carried out a transcriptional and karyotypic analysis of UCB derived VSEL cells. A carefully controlled cytometric analysis showed the CXCR4+ VSEL cells to comprise the largest part of the CD45^−^Lin^−^ population in hUCB, but to essentially lack the previously ascribed stem cell markers CD34, CD133 or SSEA-4. The purified human VSEL cells respond to none of a range of culture conditions known to support other stem cell populations, with the exception of a very low frequency of hematopoietic activity most probably resulting from low-level carryover of CD34^+^CD45^dim^ cells. Furthermore, microarray transcription analysis revealed VSEL cells to be clearly distinct both from adult and embryonic stem cell populations and from mature hematopoietic lineages. Last but not least, we found hVSEL cells to display a high degree of aneuploidy. These data support neither a classical adult nor embryonic stem cell like phenotype for hVSEL cells.

## Materials and Methods

Unless stated otherwise, all chemicals were purchased from Sigma-Aldrich, Taufkirchen, Germany.

### Preparation of primary human umbilical cord blood cells

Human UCB was obtained from healthy full-term pregnancies with informed written consent of the parents according to guidelines approved by the ethics committee of the Universität Leipzig and according to the manufacturing authorisation for UCB transplants of Vita34 AG. Fresh cord blood was analyzed within 48 hours after birth. Total nucleated cells (TNC) were prepared by ammonium chloride lysis (lysis buffer: 150 mM NH_4_Cl, 10 mM NaHCO_3_, 0,1 mM EDTA; 1–2×10 min, room temperature (RT)). Light-density mononuclear cells (MNC) were isolated from the interphase after Ficoll density gradient centrifugation (35 min; 300 g; Ficoll 1.077 kg/L, Biochrom, Berlin, Germany) and also subjected to ammonium chloride lysis. High-density nucleated cells, which separated together with erythrocytes in the pellet under the Ficoll gradient, were also recovered by ammonium chloride lysis. After lysis, all cells were washed with PBS with 0.5% human serum albumin (HSA, PAA, Pasching, Austria) and 0.6% citrate phosphate dextrose (CPD). This buffer was also used for subsequent antibody labelling, FACS analysis, and cell sorting.

### FACS analysis

Multiparameter FACS analysis was conduced on an LSR II flow cytometer with DIVA 6.1.3 software (Becton Dickinson, Heidelberg, Germany). FlowJo 9 software (Tree Star Inc., Ashland, USA) was used for data evaluation. Monoclonal antibodies were purchased from Becton Dickinson, Heidelberg, Germany (SSEA-1-phycoerythrin (PE), (MC480); SSEA-3-PE (MC631); SSEA-4-PE (MC813-70); Tra-1-81-Alexa 647 (Tra-1-81); CD140a-PE (αR1); CD184-PE (12G5); CD235a-PE-Cy5 (HIR2)); Biolegend, Fell, Germany (CD31-Alexa 488 (WM59); CD34-PE-Cy7 (581); CD48-PE (BJ40); CD324-allophycocyanin (APC), (67A4); eBioscience, Frankfurt, Germany (CD38-APC (HIT2); CD45-Biotin and CD45-APC-eFlour 780 (HI30); CD84-Biotin (2G7); CD90-PE-Cy5 (5E10); CD105-Alexa 488 (43A3); CD144-APC (16B1); CD243-PE (UIC2); CD244-PE (C1.7)); Miltenyi Biotec, Bergisch Gladbach, Germany (anti-biotin-fluorescein isothiocyanate (FITC), (Bio3-18E7); FcR-Blocking Reagent human (#130-059-901); Lineage Cell Depletion Kit (#130-092-211) incl. CD2, CD3, CD11b, CD14, CD15, CD16, CD19, CD56, CD123 and CD235a; MSCA-1-Biotin (W8B2); PSA-NCAM-PE (2-2B); CD133-APC (293C3); CD271-APC (ME20.4-1.H4)); and R&D Systems, Wiesbaden-Nordenstadt, Germany (CD338-PE (3D5)). All antibody stainings were conducted for 20–30 min at RT in the presence of FCR-Blocking Reagent. The cell permeable DNA dye Hoechst 33342 (HO) was included at 10 µg/mL during the antibody staining to allow the discrimination of nucleated cells (HO^+^) from cell debris (HO^−^). 2 µg/mL propidium iodide (PI) was added to the FACS tube before analysis to allow the exclusion of PI^+^ dead cells.

### Cell sorting

Lineage negative (Lin^−^) cells were prepared from TNC using the Lineage Cell Depletion Kit with LS columns according to the manufacturer's instructions (Miltenyi Biotec, Bergisch Gladbach, Germany). Lin^−^ cells were first labelled with anti-CD45-biotin, followed by a secondary staining with anti-biotin-FITC, anti-CXCR4-PE and anti-CD34-PE-Cy7. Stainings were conducted at RT for 20–30 min. Before FACS sorting, PI was added at 2 µg/mL to allow the exclusion of dead cells. In a first round of FACS sorting, Lin^−^CD45^dim^CD34^+^ cells, and Lin^−^CD45^−^ cells were enriched. A second sort of the separated populations was conducted to further purify CD34^+^CD45^dim^ cells and Lin^−^CD45^−^CXCR4^+^ VSEL cells, respectively. For microarray analysis, Lin^−^CD45^−^CXCR4^+^CD34^−^ (CD34^−^VSEL cells) and Lin^−^CD45^−^CXCR4^+^CD34^+^ (CD34^+^VSEL cells) were separated during the second sort.

### Cytometry and cell staining

Cytospins from sorted CD34^+^CD45^dim^ cells or VSEL cells were air dried, fixed with methanol and stained with May-Grünwald (5 min) and Giemsa (1∶20, 20 min). Cytospin preparation leads to flattening of larger cells. Hence, the size of CD34^+^CD45^dim^ cells and VSEL cells was determined from sorted cells that were allowed to sediment onto poly-L-lysin coated slides. The area and diameter of CD34^+^CD45^dim^ cells and VSEL cells immobilised that way was measured manually using NIS-Elements Br Software Package (Nikon, Düsseldorf, Germany).

Karyotyping of cytospin preparations was done with site specific fluorescent in situ hybridisation (FISH) probes from Metasystems (Altlussheim, Germany) according to the instruction of the manufacturer. The dual-colour kit XL P53/DLEU detects the long arm of chromosome 13 and in the short arm of chromosome 17. A Texas Red labelled probe detects a specific region at 13q14 including the DLEU1 and DLEU2 genes and marker D13S319. The FITC labelled probe hybridizes specifically to the TP53 gene at 17p13. The dual-colour centromeric probe kit XCE 7/8 was used to identify chromosomes 7 and 8. The probe for chromosome 7 was labelled with Texas Red, the probe for chromosome 8 with FITC.

### Cell culture

Basic cell culture media, human serum albumin (HSA), trypsin, accutase, L-glutamine, and leukemia inhibitory factor (LIF) were purchased from PAA (Pasching, Austria). Human recombinant cytokines (stem cell factor (SCF), thrombopoietin (TPO), Fms-related tyrosine kinase 3 ligand (FL), epidermal growth factor (EGF), fibroblast growth factor-basic (FGF), tumour growth factor-beta (TGF-β)) were purchased from Peprotech (Hamburg, Germany). CHIR99021 (GSK-3β inhibitor) was purchased from Sigma-Aldrich (Taufkirchen, Germany). Commercial media for human hematopoietic stem cells (Methocult, Stem Cell Technologies, Köln, Germany), human pluripotent stem cells (Nutristem, Stemgent/Miltenyi, Bergisch Gladbach, Germany; TeSR1, Stem Cell Technologies, Köln, Germany), murine pluripotent stem cells (ESGRO, Millipore, Schwalbach, Germany) and mesenchymal stem cells (Stempro MSC SFM, Invitrogen, Karlsruhe, Germany; MesenchymStem Medium, PAA, Pasching, Austria) were used according to the instruction of the manufacturer. Cultures with media for pluripotent stem cells were set up in culture plates coated with Matrigel (BD, Heidelberg, Germany). OP9 stroma cells were cultured in Iscove's modified dulbecco's medium (IMDM) with 20% fetal calf serum (FCS), 2 mM L-glutamine. Mouse embryonic fibroblasts (MEF) were cultured in Dulbecco's modified eagle medium (DMEM) low glucose with 1% non-essential amino acids and 10% FCS. Freshly sorted VSEL cells were cultured in various cell culture media including all commercial media and self-made media supplemented with combinations of SCF, FL and TPO; EGF and FGF; LIF; and FGF and TGF-β each with and without CHIR99021. VSEL cells were also seeded onto OP9 or irradiated MEF stroma cells.

### Microarray analysis

Sorted CD34^−^ and CD34^+^VSEL cells from three individual cord blood units were lysed in RLT buffer (Qiagen, Hilden, Germany) and stored at −80°C. RNA extraction, labelling, hybridisation and microarray analysis was conducted by AROS Applied Biotechnology AS (Aarhus, Denmark) using Rneasy kit (Qiagen, Hilden, Germany), NuGen Pico labelling kit (NuGen, San Carlos, USA) and Affymetrix GeneTitan Human Genome U219 microarray (Affymetrix, Santa Clara, USA) according to in-house protocols. Microarray data quality was checked with arrayQualityMetrics [16].

Our transcription data were compared with >200 raw datasets from the gene expression omnibus (GEO) database using R, bioconductor and MultiExperiment Viewer (MeV) according to the following algorithm [17], [18]. Briefly, [I] raw data were loaded into R, [II] probe sets for each platform were annotated, [III] genes, proteins or transcripts common to all platforms were matched, including checks for redundancy or missing values, [IV] data were compiled into a new “virtual array", [V] normalized and [VI] subjected to batch effect removal using ComBat.R with MAQC (microarray quality control) data sets as an internal control for every platform [19]. The algorithm has been implemented into a new bioconductor package called virtualArray (http://www.bioconductor.org/packages/2.10/bioc/html/virtualArray.html).

The “virtual array" thus generated was exported and analyzed in MeV. Transcriptome wide principal component analysis (PCA) and hierarchical clustering (HCL) were performed to illustrate similarities and differences between VSEL cells and embryonic and adult stem cells, as well as mature blood cell types [20], [21]. An un-rooted cluster tree was generated with Archaeopteryx [22]. Furthermore, a more focused comparison of a potential pluripotency signature in VSEL cells was conducted using a subset of pluripotency associated genes queried from www.genecards.org (search term: pluripotency; category: protein-coding) [23]. 371 of 413 queried genes were retrieved in the virtual array and used for further evaluation (**Table S1**). Expression levels in selected cell populations were tested for significance by Mann-Whitney test (critical p-value = 0.01).

### Statistics and graphs

Statistical analysis and box-plots were performed with Analyse-it (Analyse-it Ltd., Leeds, United Kingdom). Skeletal box-plots show median, 1^st^ and 3^rd^ quartile, maximum and minimum. Student's t-test and the Mann-Whitney test and were used to test significance where appropriate.

## Results

### The immunophenotype of CD45^−^Lin^−^ hUCB cells

**Figure 1:**
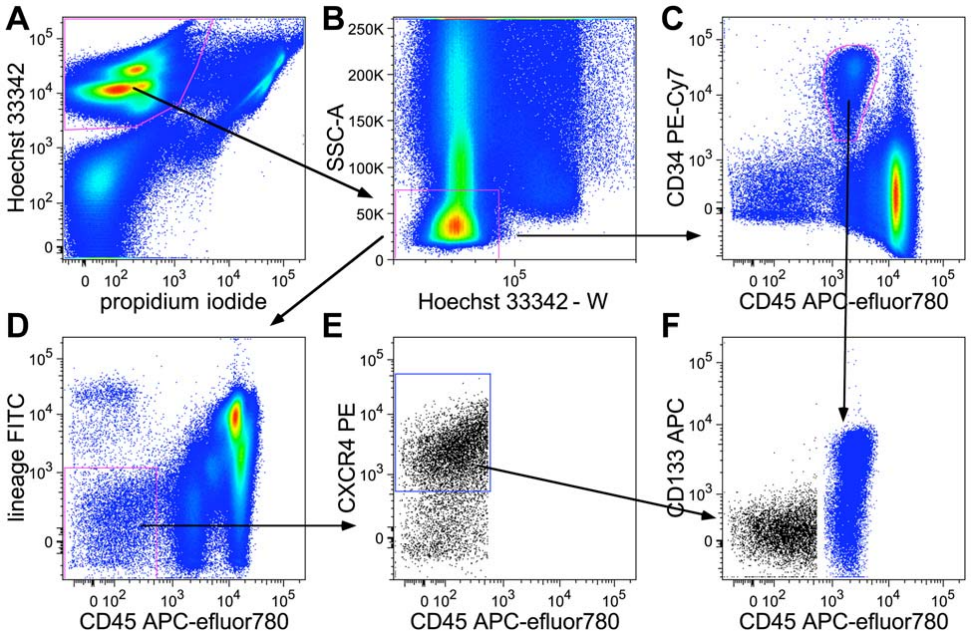
Gating of VSEL cells and CD34+CD45dim cells in hUCB. hUCB TNC were isolated by NH_4_Cl lysis and stained with fluorescent antibodies, Hoechst 33342, and PI. A) Gating of living (PI^−^) nucleated (Hoechst 33342^+^) cells; B) pulse processing: exclusion of doublets (Hoechst-W^high^) and granulocytes (SSC-A^high^). C) Gating of CD34^+^CD45^dim^ Hoechst 33342^+^PI^−^. D) Gating of CD45^−^Lin^−^ cells. E) Gating of CD45^−^Lin^−^ CXCR4^+^Hoechst 33342^+^PI^−^ VSEL cells. F) Expression of CD133 in CD34^+^CD45^dim^ cells (blue) and VSEL cells (black).

Human umbilical cord blood derived VSEL cells have been described to be CD45^−^Lin^−^ and to carry one or more of the cell surface markers CXCR4, CD133, CD34 and SSEA-4. However, none of these markers is unique for VSEL cells, as they are also expressed on hematopoietic stem and progenitor cells (CD34, CD133), leucocytes (CXCR4) or erythroid cells (SSEA-4). Aiming to define the VSEL cell phenotype more thoroughly, we selected a number of established markers for embryonic, mesenchymal, hematopoietic and neuronal stem cells, and analysed their abundance and distribution on the population of living nucleated CD45^−^Lin^−^ cells, of which the VSEL population is a part. The exclusion of dead cells and cell debris by PI and Hoechst 33342 staining, the reduction of false positive events by FcR-blocking reagent, and the exclusion of doublets by pulse processing the FACS signals proved vital to the clear discrimination and gating of CD45^−^Lin^−^ cells in UCB preparations after NH_4_Cl lysis (**Figure 1**).

**Figure 2:**
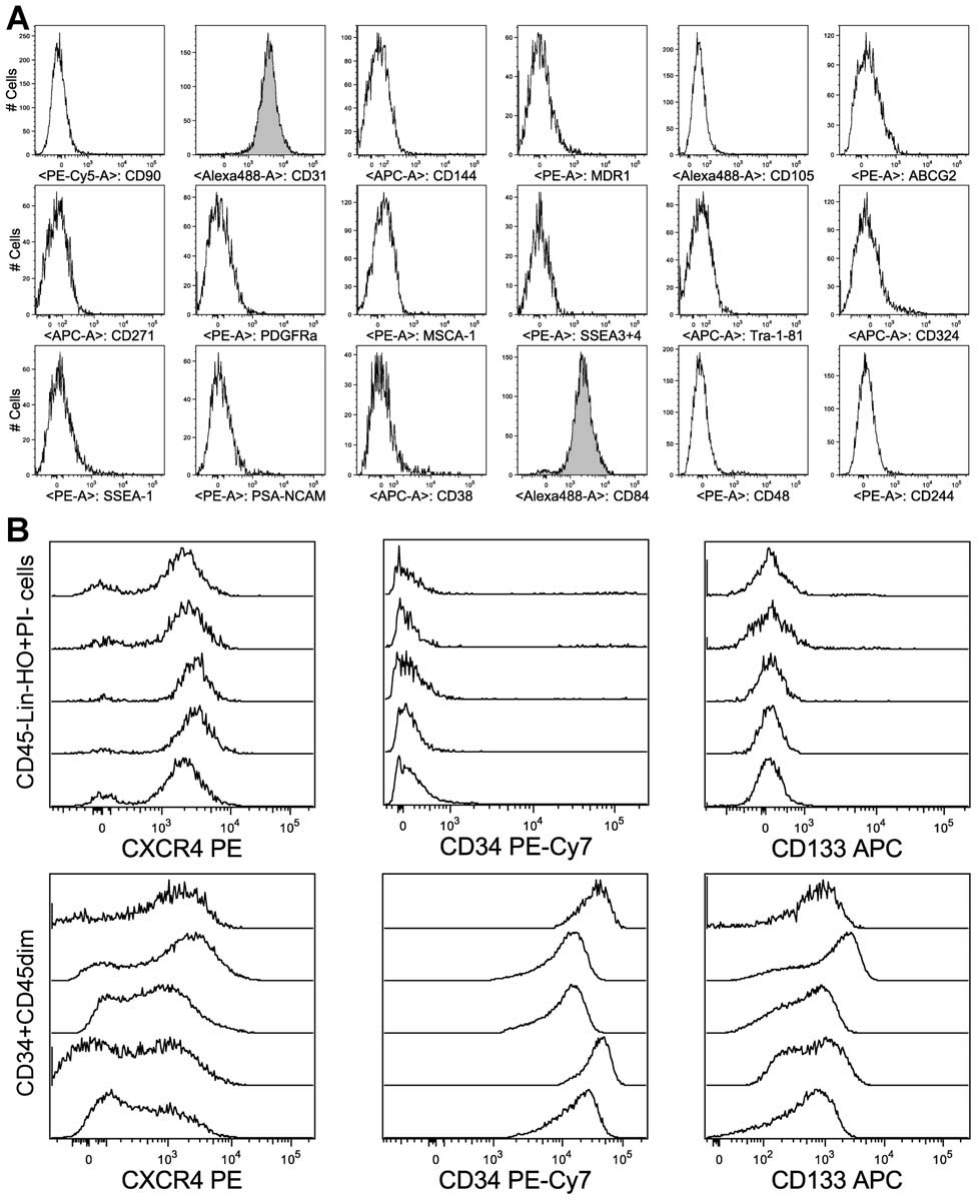
Immunophenotype of CD45-Lin-Hoechst 33342+PI- cells. hUCB cells were subjected to multiparameter FACS analysis, and gated according to **Figure 1** D (either Lin^−^ or CD235a^−^). Among 22 markers tested, three were found positive on CD45^−^Lin^−^ cells: CXCR4, CD31 and CD84. However, in contrast to previous reports, CD45^−^Lin^−^ cells contain very low amounts of either CD34^+^ (1,2%+/−0,6%; n = 5) or CD133^+^ (1,8%+/−2,3%; n = 5) cells (B middle & right). A) Representative single analysis of markers on CD45^−^Lin^−^ cells. B+C) Representative replicate analysis of the expression of CXCR4, CD34 and CD133 on CD45^−^Lin^−^ cells and on CD34^+^CD45^dim^cells.

From a total of 22 markers tested, only three were found positive: CD31, CD84 and the previously described VSEL cell marker CXCR4 (**Figure 2**, **Table 1**). All of these markers were >90% positive on the CD45^−^Lin^−^ population. In addition to confirming that the cells identified here are equivalent to those originally described as VSEL by Kucia et al. [8], this shows that the CD45^−^Lin^−^ population is essentially identical to the previously described CD45^−^Lin^−^CXCR4^+^ VSEL cell population, provided cell debris, dead cells and doublets are identified and gated-out during data analysis.

**Table 1:** Immunophenotyping and transcriptional profiling of Lin-CD45- and VSEL cells.

Marker	Synonyms	Typically present in tissue or stem cell	Present in Lin-CD45- cells and subpopulations			Ref.
			total#)	CXCR4^+^CD34^−^ *)	CXCR4^+^CD34^+^ *)	
MSCA-1	TNAP	MSC	−	−	−	[13]
SSEA-3		ESC	−	n/a	n/a	[34]
SSEA-4		ESC; MSC; erythrocytes	−	n/a	n/a	[34]–[36]
Tra-1-81	PODXL	ESC	−	−	−	[34]
CD15	SSEA-1; FUT4	murine ESC; granulocytes; monocytes	−	−	−	[34], [37]
CD31	PECAM-1	EPC; mature and primitive hematopoietic cells	+	+	+	[38]–[40]
CD34		HSPC; EPC	−	−	−	[41], [42]
CD38		leukocytes; -ve on primitive human HSPC	−	−	−	[43]
CD48	SLAMF2	lymphocytes; -ve on murine HSPC	−	−	−	[44]
CD56(PSA)	PSA-NCAM	NSC	−	−	−	[45]
CD84	SLAMF5	most mature and primitive hematopoietic cells	+	+	+	[46]
CD90	Thy-1	HSPC	−	−	−	[47]
CD105	endoglin; SH-2	HSPC; MSC	−	−	−	[48], [49]
CD133	prominin-1	HSPC; NSC; EPC;	−	−	−	[50]–[52]
CD140a	PDGF-Ra	murine MSC	−	−	−	[33]
CD144	VE-cadherin	EPC	−	−	−	[38]
CD184	CXCR4; fusin	most mature and primitive hematopoietic cells	+	+	+	[8], [53]
CD243	MDR-1; p-gp	reported in SP cells; blood-brain barrier	−	−	−	[54]
CD244	SLAMF4	NK-cells; -ve on murine HSPC	−	−	−	[44]
CD271	p75(NTR)	MSC; NSC	−	−	−	[13], [55]
CD324	E-cadherin	ESC; various tissues	−	−	−	[56]
CD338	ABCG2; BCRP-1	reported in SP cells; placenta	−	−	−	[57], [58]

#)surface antigen expression measured by FACS analysis;

*)mRNA expression level measured by microarray analysis.

CD31 and CD84 emerge from this analysis as two newly identified positive markers on VSEL cells. However, CXCR4, CD31 and CD84 are all also present on various types of mature blood cells and therefore cannot be used alone or in combination for stringent positive enrichment of VSEL cells. Somewhat surprisingly, very few CD133 or CD34 were detected on the CD45^−^Lin^−^ population even when up to 2*10^6^ TNC were analysed (**Figure 2 B**).

### Loss of VSEL cells during ficoll gradient centrifugation

**Figure 3:**
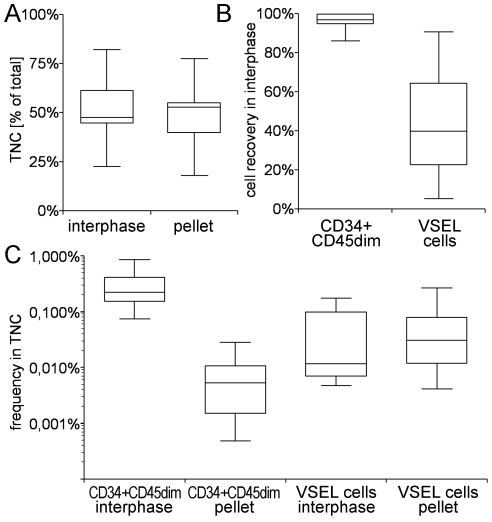
Separation of CD34+CD45dim cells and VSEL cells by ficoll density gradient centrifugation. Cells recovered from interphase and pellet were lysed with NH_4_Cl and analyzed for the frequency of CD34^+^CD45^dim^ cells and CD45^−^Lin^−^CXCR4^+^ VSEL cells, respectively. A) Recovery of TNC in interphase and pellet (n = 15). B) Recovery of CD34^+^CD45^dim^ cells and VSEL cells in the interphase (n = 15). C) Frequency of CD34^+^CD45^dim^ cells and VSEL cells in interphase and pellet, respectively (n = 15).

It has been previously reported that ficoll density gradient centrifugation commonly used to recover mononuclear cells (MNC) and CD34^+^CD45^dim^ cells from UCB leads to loss of VSEL cells [8], [15], [24]. To investigate this phenomenon in more detail, and to determine whether or not VSEL cells could be recovered from the pellet after density gradient centrifugation, we have studied the distribution of VSEL cells and CD34^+^CD45^dim^ cells between the interphase and the pellet using standard ficoll (1.077 kg/L) density gradients. After separation and subsequent ammonium chloride lysis, we were able to recover roughly equal amounts of TNC from the interphase (predominantly mononuclear cells) and pellet (predominantly erythrocytes and polymorpholeukocytes) (**Figure 3 A**). As expected, CD34^+^CD45^dim^ cells were predominantly enriched in the interphase. In contrast, VSEL cells were more abundant in the pellet, indicating that the density of most VSEL cells is higher than that of CD34^+^CD45^dim^ cells and mononuclear cells (**Figure 3 B, C**). Thus, while VSEL cells are insensitive to NH_4_Cl lysis, we confirm that ficoll density gradient centrifugation does indeed lead to considerable loss of ∼60% (10–95%; n = 15) of the VSEL cells. Since we did not detect any phenotypic differences between VSEL cells from the interphase or the pellet during subsequent FACS analysis, we chose to pre-enrich by NH_4_Cl lysis rather than density gradient separation in subsequent experiments in order to retain the entire VSEL population.

### Isolation and purification of VSEL cells

**Figure 4:**
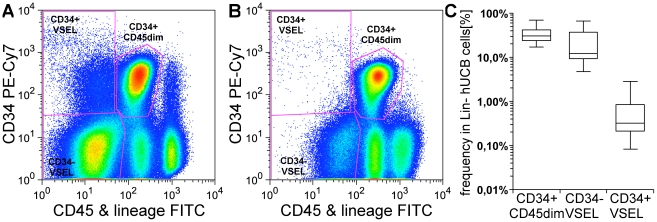
FACS sorting of VSEL cells from Lin− hUCB cells derived from MACS lineage depletion of TNC. A+B) Two extreme UCB samples with many (A) and very few (B) CD34^+^VSEL cells, respectively. C) Frequency of CD34^+^CD45^dim^ cells (upper middle gate in A+B), CD34^−^VSEL (CD45^−^Lin^−^CXCR4^+^CD34^−^, lower left gate in A+B), and CD34^+^VSEL (CD45^−^Lin^−^CXCR4^+^CD34^+^, upper left gate in A+B) cells within PI^−^ lymphocytes (n = 14).

In an average hUCB less than 1 in 5000 TNC is a CD45^−^Lin^−^CXCR4^+^ VSEL cell. Since direct sorting of such low frequency cells is associated with increased sort times and a decrease in yield and purity, we chose a strategy of MACS based lineage depletion followed by double FACS sorting for high purity. In this way, we were able to successfully isolate a population of CD45^−^Lin^−^CXCR4^+^ cells from every hUCB tested. In order to better distinguish the CD45^−^ VSEL cells from the CD45^dim^ cells in FACS plots, we also stained for CD34, which the analyses shown above show to be highly expressed on CD45^dim^ cells but very rare on VSEL cells. At the higher level of resolution possible after removing Lin^+^ TNC, we did detect a very low level of CD34^+^VSEL cells in some UCB samples (**Figure 4**). Although these account for a relatively small fraction of the VSEL population, we selected three hUCB samples with prominent CD34^+^VSEL cells and separated CD34^+^ and CD34^−^ VSEL cells for the individual transcriptional profiling described below. In all other experiments, we pooled the CD34^+^VSEL cells into the much larger CD45^−^Lin^−^CXCR4^+^CD34^−^ population. The averaged frequency of CD34^+^CD45^dim^ cells, CD34^−^VSEL and CD34^+^VSEL cells in lineage negative UCB cells was 33%+/−14%, 22%+/−19%, and 0.76%+/−0.96%, respectively (n = 14, mean +/− SD).

### In vitro culture of VSEL cells

The ability to expand and/or differentiate in vitro under appropriate culture conditions is a common property of stem and progenitor cells of different origins and has been reported for murine VSEL cells. We entered hUCB derived VSEL cells into a range of different culture media used to expand adult or pluripotent stem and progenitor cells. These included stroma supported culture (MEF, OP9), stroma-free culture in our own media supplied with a range of different cytokines (SCF, TPO, FL, LIF, EGF, FGF, TGF-beta) or small molecules (CHIR99021) and commercial media prepared specifically to support hematopoietic, mesenchymal or pluripotent stem cells (Methocult, Stempro MSC SFM, Nutristem, TeSR1, ESGRO, MesenchymStem).

None of the media tested supported VSEL cell proliferation in vitro. A low frequency of hematopoietic colonies were detected as cobblestone areas giving rise to non adherent mature hematopoietic cells on OP9 stroma. However, since such colonies arose at a >100 fold higher frequency from the CD34^+^CD45^dim^ population, the few colonies originating from the neighbouring CD45^−^VSEL population are very likely to be due to carry over of a low frequency of haematopoietic progenitor cells.

### Transcriptional profile

Transcriptional profiling of purified VSEL cells was performed by Affymetrix microarray analysis. For this study, we chose three individual blood samples with prominent CD34^+^VSEL cells and included both CD45^−^Lin^−^CXCR4^+^CD34^−^ (CD34^−^VSEL) and CD45^−^Lin^−^CXCR4^+^CD34^+^ (CD34^+^VSEL) cells separately in the analysis. The mRNA profile revealed a high degree of similarity between the CD34^−^ and CD34^+^VSEL populations, with no indication of one population being more immature than the other. However, we found close accordance between the surface antigens measured by FACS analysis and the transcription level of their corresponding genes measured by microarray (**Table 1**). The only deviation was the level of CD34 mRNA in CD34^+^VSEL cells, which was both above the median and higher than in CD34^−^VSEL cells, but below the set threshold (**Figure S1**).

**Figure 5:**
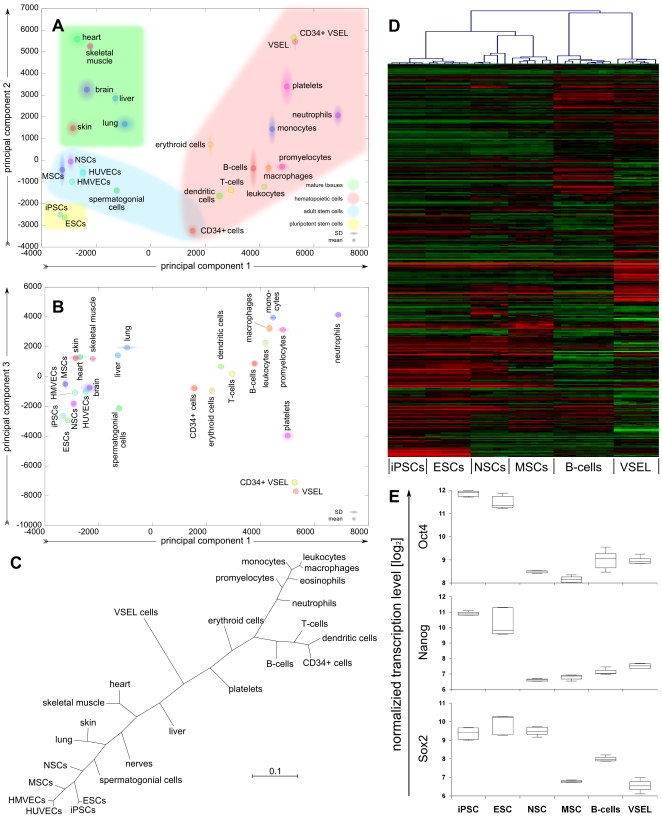
Microarray analysis of sorted hUCB VSEL cells and comparison to other stem and mature cell types (listed in Table S2). A+B) A genome-wide unbiased principal component analysis including 12621 genes classifies VSEL cells as remote to both pluripotent and adult stem cell populations as well as mature blood cell lineages. C) Hierarchical clustering represented by an un-rooted tree diagram confirms the results of the PCA. D) A heatmap comparison of 371 selected pluripotency associated genes shows substantial differences between VSEL cells and pluripotent stem cells, but a high degree of similarity between ES and iPS cells. E) Normalized transcription level of the core pluripotency genes Oct4 (POU5F1), Nanog and Sox2 as part of D. Note that the Affymetrix microarray probes for POU5F1 do not comply with up to date quality standards as they are known to detect pseudogenes.

We classified our data sets in respect to more than 200 published transcription data sets from the GEO database to compare the transcriptional profile of VSEL cells to those of previously characterised, well-defined populations of pluripotent stem cells, adult stem cells and mature blood cell types (**Table S2**). **Figure 5** shows the results of three different approaches. Firstly, a transcriptome-wide principal component analysis including over 12000 genes revealed VSEL cells to be clearly distinct both from all of the known stem cell populations and from the mature hematopoietic lineages tested (**Figure 5 A+B**). The PCA illustrates a developmental hierarchy ranging from the most immature pluripotent stem cells (lower left) to mature hematopoietic lineages (upper right). Considering PC1 and PC2, the transcriptional signature of VSEL cells appears closest to the hematopoietic lineage. However, PC 3 illustrates that VSEL cells are dissimilar to any of the mature hematopoietic cell types tested. Furthermore, no similarity was observed to any of the mature tissue samples included, and VSEL cells were clearly far removed from pluripotent stem cells. A similar result was obtained by hierarchical clustering (HCL) as shown in **Figure 5 C**. The un-rooted tree diagram thus derived shows two main branches: hematopoietic cells, and non-hematopoietic cells and tissues including adult and pluripotent stem cells. VSEL cells locate as a third, independent branch remote from all other cell types. Finally, we focused on the transcription of 371 selected pluripotency associated genes to investigate more directly the relationship between VSEL and pluripotent stem cells. **Figure 5 D** shows that the transcriptional profile of these genes is highly similar in embryonic stem cells (ESC) and induced pluripotent stem cells (iPS). Furthermore, neuronal stem cells (NSC), mesenchymal stromal cells (MSC), mature B-cells and VSEL cells are clearly distinct from pluripotent stem cells, VSEL cells and B-cells being most dissimilar and remote to ESC. Oct4, Nanog and Sox2 were highest in iPS and ES cells with an expectedly high level of Sox2 transcripts also in NSCs (**Figure 5 E**). In summary, the transcriptional analysis confirm the absence of stemness markers in VSEL cells, but reveal a unique transcriptional signature with little similarities to other stem cell or mature blood cell types.

### VSEL cells show an aberrant karyotype and possess hardly any cytoplasm

**Figure 6:**
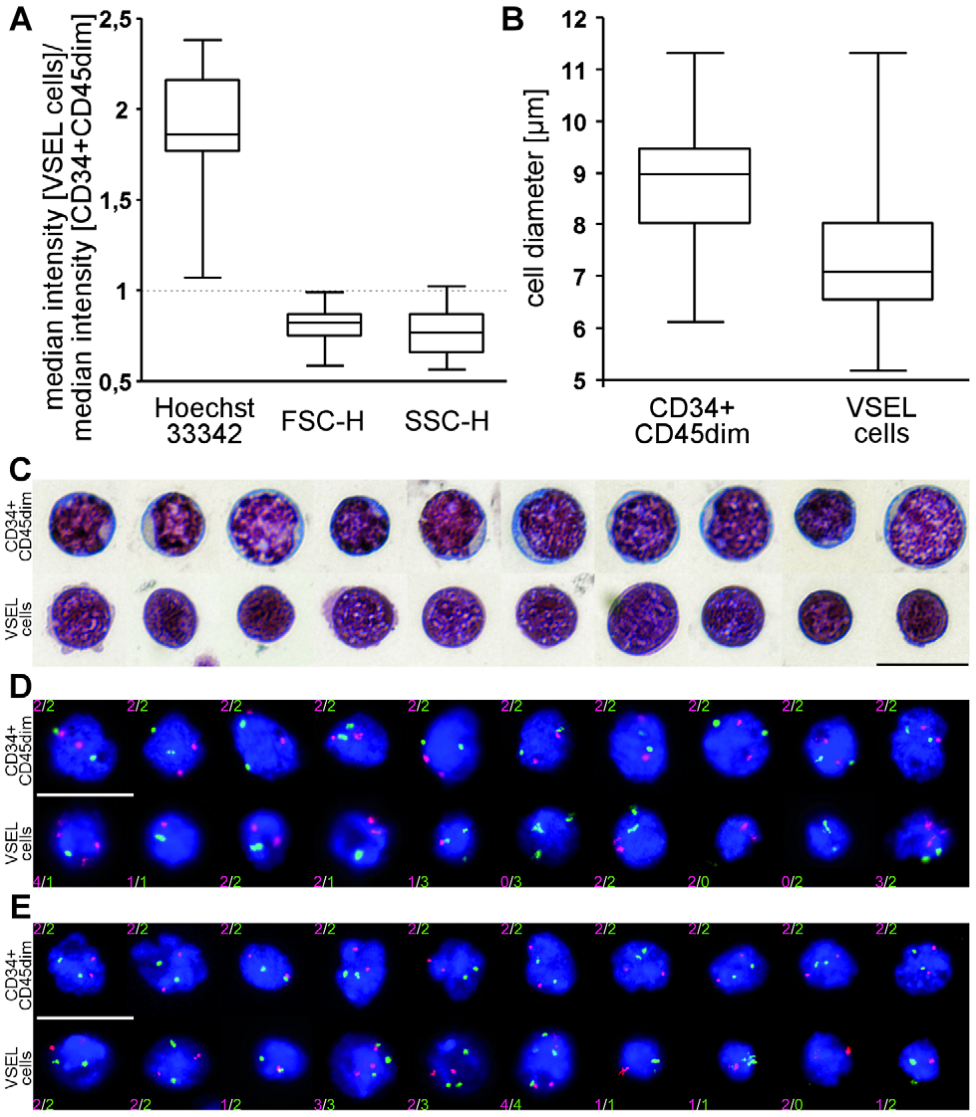
Cytometric and karyotypic analysis of CD34+CD45dim cells and VSEL cells. A) Comparison of the median Hoechst 33342 fluorescence, FSC-H and SSC-H of CD34^+^CD45^dim^ cells and VSEL cells, respectively (n = 15); B) Cell diameter of freshly FACS sorted CD34^+^CD45^dim^ cells and VSEL cells (n≥110; p<0.001); C) May-Grünwald/Giemsa stained cytospin preparations of CD34^+^CD45^dim^ cells (top) and VSEL cells (bottom). D–E) Karyotypic analysis of cytospin preparations of CD34^+^CD45^dim^ cells (top) and VSEL cells (bottom) stained with probes against D) centromere 7 (red) and centromere 8 (green), and E) p53 (red) and DLeu (green). scales = 10 µm.

Our ficoll density gradient experiments and FACS analysis data confirmed a relatively high density and small size of VSEL cells compared to CD34^+^CD45^dim^ cells (**Figure 1**). Furthermore, direct measurement of FACS sorted CD45^−^Lin^−^CXCR4^+^ VSEL and CD34^+^CD45^dim^ cells showed them to have diameters of 7.1 µm vs. 9.0 µm repectively (p<0.0001; **Figure 6 B**), which is in close agreement with the size of CD45^−^Lin^−^CXCR4^+^ cells (7.4 µm) measured by ImageStream analysis by Zuba-Surma et al. [15]. Surprisingly, despite this smaller size, a semi-quantitative estimation of the DNA content using the cell permeable dye Hoechst 33342 showed an approximately 2-fold higher fluorescence of VSEL cells compared to mononuclear CD34^+^CD45^dim^ cells. This suggested either binuclearity, tetraploidy, or an unusual chromatin conformation that results in a significantly higher intercalation of Hoechst into the DNA of VSEL cells (**Figure 6 A**). To investigate this further, we directly analysed highly purified VSEL cells in cytospin preparations. **Figure 6 C** highlights the clear morphological differences between VSEL cells and CD34^+^CD45^dim^ cells. Compared to CD34^+^CD45^dim^ cells, VSEL cells are not only smaller, but appear to possess barely any cytoplasm and a less structured nucleoplasm, consistent with the previous description by Kucia et al. [8]. In order to clarify the ploidy status with respect to the higher Hoechst 33342 fluorescence, we used four different locus specific interphase FISH probes for chromosomes 7, 8, 13 and 17. To our surprise, we found that the majority of VSEL cells displayed an aberrant karyotype, even with this limited number of markers. While CD34^+^CD45^dim^ cells generally displayed the expected diploid karyotype with two signals from each of the probes used, VSEL cells showed substantial deviations from the normal, ranging from 0–4 signals per probe and cell (**Figure 6 D, E**). Thus, the high Hoechst 33342 fluorescence in VSEL cells results not from a tetraploid, but from an aberrant karyotype.

## Discussion

Very small embryonic like stem cells were isolated firstly from murine bone marrow and later from human umbilical cord blood as a rare population of small CD45^−^Lin^−^CXCR4^+^ cells with a high nucleus to cytoplasm ratio [8], [9]. Our independent characterization of CD45^−^Lin^−^CXCR4^+^ VSEL cells from hUCB confirms that they are indeed significantly smaller than CD34^+^CD45^dim^ hematopoietic cells, possess hardly any cytoplasm and express both CD31 and CD84, but calls into question their stem cell-like nature.

The identification and isolation of VSEL via FACS techniques proved to be reliable and reproducible only after the adoption of a number of measures to reduce non-specific signals, including the exclusion of debris and dead cells on the basis of Hoechst 33342 and PI staining. The resulting populations were almost uniformly positive for CXCR4, CD31 and CD84, but contained low to undetectable amounts of CD133, CD34 and most adult and embryonic stem cell markers. This pattern of surface antigen expression was supported by transcriptional profiling of the highly purified VSEL population, but contrasts with previous reports that CD34 antigen is detectable on a higher frequency of VSEL cells than is CXCR4 [8].

The transcriptional profiling also permitted comparison of VSEL cells to published data covering a range of primitive and mature blood cell types as well as a panel of adult and pluripotent stem cells. This showed VSEL cells to be unique population with no clear relation to either hematopoietic cells or other stem cell types. These findings, together with the failure of hUCB VSEL to respond to any of a range of culture environments designed to support the expansion of pluripotent or adult stem cells, call into question any stem cell like character of the cells. Perhaps most strikingly, we subsequently discovered that VSEL cells are clearly aneuploid, so that using just four different probes identifies karyotypic aberrations in the majority of cells, including the complete absence of single loci.

The cumulative evidence therefore suggests strongly that hUCB VSEL cells are the products of a defective or uncoordinated cell division, leaving them with extensive karyotypic abnormalities. The associated very low cytoplasmic volume would also be consistent with the observed loss of cellular function. Although aneuploidy has long been associated with cancer, it has recently been observed in cultured pluripotent and neuronal stem cells as well as normal neuronal progenitors and primary cells from blastocysts, so that the tendency to generate aneuploid cells may be a normal feature of regenerative systems [25]–[28]. Even so, the aneuploid products themselves are unlikely to make a long term contribution to regeneration, so that the sum of the evidence presented here argues clearly against a stem cell identity of VSEL cells from hUCB.

It has previously been reported that UCB derived VSEL cells can be expanded to short term repopulating hematopoietic cells on OP9 feeder layers [29]. However, we have shown in this study that stringent separation of CD45^−^ VSEL cells from the CD45^dim^ hematopoietic progenitor and stem cell population by FACS sorting is very difficult. For this reason, we suspect that the low frequency of hematopoietic cells that we detected on OP9 stroma seeded with the CD45- VSEL population result from carry-over of hematopoietic progenitors into the VSEL gate, rather than from hematopoietic differentiation of VSEL cells. Indeed, there has been no independent evidence to date to support that a pure UCB VSEL population possesses functional hallmarks of stem cells, such as in vitro differentiation potential, high proliferative capacity or the ability to self-renew in vivo. It is therefore questionable whether UCB VSEL cells should be termed “stem cells" at all.

The properties of UCB VSEL cells reported here differ markedly from those of VSEL cells derived from murine bone marrow, which have been described similarly as putatively pluripotent, small and rare CD45^−^Lin^−^Sca-1^+^ cells. While rigorous proof of pluripotency is also lacking for murine VSEL cells, they have at least demonstrated functional adult stem cell properties in vitro [9], [30] and recent in vivo studies have shown that murine VSEL cells can form bone tissue and may contribute to heart regeneration [31], [32]. Furthermore, Morikawa et al. have identified a novel type of mesenchymal precursor (CD45^−^Lin^−^Sca-1^+^PDGFR-a^+^ “PαS" cells) within the VSEL cell population, capable of engrafting in vivo and regenerating mesenchymal tissue in the bone marrow [33]. There is therefore little doubt that the VSEL cells isolated from murine bone marrow, while being functionally heterogenous, contain very promising stem cell activities. It is to be hoped that similar cells are present in human tissues. However, our studies suggest strongly that the hUCB-derived cells described to date as VSEL cells are not the human equivalent of murine VSEL cells and should not be regarded as a stem cell population.

## Supporting Information

Figure S1 Transcript level of genes coding for selected surface antigens in CD34^−^VSEL and CD34^+^VSEL cells. Genes with a median transcript level above the threshold (3^rd^ quartile +1.5× inter quartile range (IQR)) were defined as positive, others as negative. While most transcripts are negative, CD31, CD84 and CD184 (CXCR4) are positive in both cell populations. These data agree with the flow cytometric immunophenotyping of VSEL cells. The level of CD34 mRNA in CD34^+^VSEL cells was below the set threshold, but was both the highest of all negative values and higher than in CD34^−^VSEL cell.
(TIF)

Table S1 Median transcription level of 371 selected pluripotency associated genes queried from www.genecards.org.
(DOC)

Table S2 Transcription data sets used in the microarray analysis.
(DOC)

## References

[1] D'Ippolito G, Diabira S, Howard GA, Menei P, Roos BA (2004). Marrow-isolated adult multilineage inducible (MIAMI) cells, a unique population of postnatal young and old human cells with extensive expansion and differentiation potential.. J Cell Sci.

[2] Kogler G, Sensken S, Airey JA, Trapp T, Muschen M (2004). A new human somatic stem cell from placental cord blood with intrinsic pluripotent differentiation potential.. J Exp Med.

[3] Jiang Y, Jahagirdar BN, Reinhardt RL, Schwartz RE, Keene CD (2002). Pluripotency of mesenchymal stem cells derived from adult marrow.. Nature.

[4] Conrad S, Renninger M, Hennenlotter J, Wiesner T, Just L (2008). Generation of pluripotent stem cells from adult human testis.. Nature.

[5] Lengner CJ, Welstead GG, Jaenisch R (2008). The pluripotency regulator Oct4: a role in somatic stem cells?. Cell Cycle.

[6] Check E (2007). Stem cells: the hard copy.. Nature.

[7] Ko K, Arauzo-Bravo MJ, Tapia N, Kim J, Lin Q (2010). Human adult germline stem cells in question.. Nature.

[8] Kucia M, Halasa M, Wysoczynski M, Baskiewicz-Masiuk M, Moldenhawer S (2007). Morphological and molecular characterization of novel population of CXCR4+ SSEA-4+ Oct-4+ very small embryonic-like cells purified from human cord blood: preliminary report.. Leukemia.

[9] Kucia M, Reca R, Campbell FR, Zuba-Surma E, Majka M (2006). A population of very small embryonic-like (VSEL) CXCR4(+)SSEA-1(+)Oct-4(+) stem cells identified in adult bone marrow.. Leukemia.

[10] Shin DM, Zuba-Surma EK, Wu W, Ratajczak J, Wysoczynski M (2009). Novel epigenetic mechanisms that control pluripotency and quiescence of adult bone marrow-derived Oct4(+) very small embryonic-like stem cells.. Leukemia.

[11] Timmermans F, Van Hauwermeiren F, De Smedt M, Raedt R, Plasschaert F (2007). Endothelial outgrowth cells are not derived from CD133+ cells or CD45+ hematopoietic precursors.. Arterioscler Thromb Vasc Biol.

[12] Jones EA, English A, Kinsey SE, Straszynski L, Emery P (2006). Optimization of a flow cytometry-based protocol for detection and phenotypic characterization of multipotent mesenchymal stromal cells from human bone marrow.. Cytometry B Clin Cytom.

[13] Battula VL, Treml S, Bareiss PM, Gieseke F, Roelofs H (2009). Isolation of functionally distinct mesenchymal stem cell subsets using antibodies against CD56, CD271, and mesenchymal stem cell antigen-1.. Haematologica.

[14] Kogler G, Sensken S, Airey JA, Trapp T, Muschen M (2004). A new human somatic stem cell from placental cord blood with intrinsic pluripotent differentiation potential.. JExpMed.

[15] Zuba-Surma EK, Klich I, Greco N, Laughlin MJ, Ratajczak J (2010). Optimization of isolation and further characterization of umbilical-cord-blood-derived very small embryonic/epiblast-like stem cells (VSELs).. Eur J Haematol.

[16] Kauffmann A, Gentleman R, Huber W (2009). arrayQualityMetrics–a bioconductor package for quality assessment of microarray data.. Bioinformatics.

[17] Saeed AI, Bhagabati NK, Braisted JC, Liang W, Sharov V (2006). TM4 microarray software suite.. Methods Enzymol.

[18] R_Development_Core_Team (2011). R: A Language and Environment for Statistical Computing.

[19] Li C, Rabinovic A (2007). Adjusting batch effects in microarray expression data using empirical Bayes methods.. Biostatistics.

[20] Raychaudhuri S, Stuart JM, Altman RB (2000). Principal components analysis to summarize microarray experiments: application to sporulation time series.. Pac Symp Biocomput.

[21] Eisen MB, Spellman PT, Brown PO, Botstein D (1998). Cluster analysis and display of genome-wide expression patterns.. Proc Natl Acad Sci U S A.

[22] Han MV, Zmasek CM (2009). phyloXML: XML for evolutionary biology and comparative genomics.. BMC Bioinformatics.

[23] Safran M, Dalah I, Alexander J, Rosen N, Iny Stein T (2010). GeneCards Version 3: the human gene integrator.. Database (Oxford).

[24] Bhartiya D, Shaikh A, Nagvenkar P, Kasiviswanathan S, Pethe P (2012). Very small embryonic-like stem cells with maximum regenerative potential get discarded during cord blood banking and bone marrow processing for autologous stem cell therapy.. Stem Cells Dev.

[25] Peterson SE, Westra JW, Rehen SK, Young H, Bushman DM (2011). Normal human pluripotent stem cell lines exhibit pervasive mosaic aneuploidy.. PLoS One.

[26] Peterson SE, Westra JW, Paczkowski CM, Chun J (2008). Chromosomal mosaicism in neural stem cells.. Methods Mol Biol.

[27] Vanneste E, Voet T, Le Caignec C, Ampe M, Konings P (2009). Chromosome instability is common in human cleavage-stage embryos.. Nat Med.

[28] Rehen SK, McConnell MJ, Kaushal D, Kingsbury MA, Yang AH (2001). Chromosomal variation in neurons of the developing and adult mammalian nervous system.. Proc Natl Acad Sci U S A.

[29] Ratajczak J, Zuba-Surma E, Klich I, Liu R, Wysoczynski M (2011). Hematopoietic differentiation of umbilical cord blood-derived very small embryonic/epiblast-like stem cells.. Leukemia.

[30] Kucia M, Wysoczynski M, Ratajczak J, Ratajczak MZ (2008). Identification of very small embryonic like (VSEL) stem cells in bone marrow.. Cell and Tissue Research.

[31] Taichman RS, Wang Z, Shiozawa Y, Jung Y, Song J (2010). Prospective identification and skeletal localization of cells capable of multilineage differentiation in vivo.. Stem Cells Dev.

[32] Zuba-Surma EK, Guo Y, Taher H, Sanganalmath SK, Hunt G (2011). Transplantation of expanded bone marrow-derived very small embryonic-like stem cells (VSEL-SCs) improves left ventricular function and remodelling after myocardial infarction.. J Cell Mol Med.

[33] Morikawa S, Mabuchi Y, Kubota Y, Nagai Y, Niibe K (2009). Prospective identification, isolation, and systemic transplantation of multipotent mesenchymal stem cells in murine bone marrow.. J Exp Med.

[34] Henderson JK, Draper JS, Baillie HS, Fishel S, Thomson JA (2002). Preimplantation human embryos and embryonic stem cells show comparable expression of stage-specific embryonic antigens.. Stem Cells.

[35] Battula VL, Bareiss PM, Treml S, Conrad S, Albert I (2007). Human placenta and bone marrow derived MSC cultured in serum-free, b-FGF-containing medium express cell surface frizzled-9 and SSEA-4 and give rise to multilineage differentiation.. Differentiation.

[36] Cooling LL, Kelly K (2001). Inverse expression of P(k) and Luke blood group antigens on human RBCs.. Transfusion.

[37] Nakayama F, Nishihara S, Iwasaki H, Kudo T, Okubo R (2001). CD15 expression in mature granulocytes is determined by alpha 1,3-fucosyltransferase IX, but in promyelocytes and monocytes by alpha 1,3-fucosyltransferase IV.. J Biol Chem.

[38] Allen JB, Khan S, Lapidos KA, Ameer GA (2010). Toward engineering a human neoendothelium with circulating progenitor cells.. Stem Cells.

[39] Watt SM, Gschmeissner SE, Bates PA (1995). PECAM-1: its expression and function as a cell adhesion molecule on hemopoietic and endothelial cells.. Leuk Lymphoma.

[40] El-Marsafy S, Carosella ED, Agrawal SG, Gluckman E, Mansur IG (1996). Functional role of PECAM-1/CD31 molecule expressed on human cord blood progenitors.. Leukemia.

[41] Testa NG, Dexter TM (1991). The biology of long-term bone marrow cultures and its application to bone marrow transplantation.. Curr Opin Oncol.

[42] Berenson RJ, Andrews RG, Bensinger WI, Kalamasz D, Knitter G (1988). Antigen CD34+ marrow cells engraft lethally irradiated baboons.. J Clin Invest.

[43] Bhatia M, Wang JC, Kapp U, Bonnet D, Dick JE (1997). Purification of primitive human hematopoietic cells capable of repopulating immune-deficient mice.. ProcNatlAcadSciUSA.

[44] Kiel MJ, Yilmaz OH, Iwashita T, Terhorst C, Morrison SJ (2005). SLAM family receptors distinguish hematopoietic stem and progenitor cells and reveal endothelial niches for stem cells.. Cell.

[45] Doetsch F, Garcia-Verdugo JM, Alvarez-Buylla A (1997). Cellular composition and three-dimensional organization of the subventricular germinal zone in the adult mammalian brain.. J Neurosci.

[46] Zaiss M, Hirtreiter C, Rehli M, Rehm A, Kunz-Schughart LA (2003). CD84 expression on human hematopoietic progenitor cells.. Exp Hematol.

[47] Murray L, Chen B, Galy A, Chen S, Tushinski R (1995). Enrichment of human hematopoietic stem cell activity in the CD34+Thy-1+Lin- subpopulation from mobilized peripheral blood.. Blood.

[48] Barry FP, Boynton RE, Haynesworth S, Murphy JM, Zaia J (1999). The monoclonal antibody SH-2, raised against human mesenchymal stem cells, recognizes an epitope on endoglin (CD105).. Biochem Biophys Res Commun.

[49] Pierelli L, Bonanno G, Rutella S, Marone M, Scambia G (2001). CD105 (endoglin) expression on hematopoietic stem/progenitor cells.. Leuk Lymphoma.

[50] Quirici N, Soligo D, Caneva L, Servida F, Bossolasco P (2001). Differentiation and expansion of endothelial cells from human bone marrow CD133(+) cells.. Br J Haematol.

[51] Koehl U, Zimmermann S, Esser R, Sorensen J, Gruttner HP (2002). Autologous transplantation of CD133 selected hematopoietic progenitor cells in a pediatric patient with relapsed leukemia.. Bone Marrow Transplant.

[52] Uchida N, Buck DW, He D, Reitsma MJ, Masek M (2000). Direct isolation of human central nervous system stem cells.. Proc Natl Acad Sci U S A.

[53] Loetscher M, Geiser T, O'Reilly T, Zwahlen R, Baggiolini M (1994). Cloning of a human seven-transmembrane domain receptor, LESTR, that is highly expressed in leukocytes.. J Biol Chem.

[54] Bunting KD, Zhou S, Lu T, Sorrentino BP (2000). Enforced P-glycoprotein pump function in murine bone marrow cells results in expansion of side population stem cells in vitro and repopulating cells in vivo.. Blood.

[55] Bixby S, Kruger GM, Mosher JT, Joseph NM, Morrison SJ (2002). Cell-intrinsic differences between stem cells from different regions of the peripheral nervous system regulate the generation of neural diversity.. Neuron.

[56] Li L, Wang S, Jezierski A, Moalim-Nour L, Mohib K (2010). A unique interplay between Rap1 and E-cadherin in the endocytic pathway regulates self-renewal of human embryonic stem cells.. Stem Cells.

[57] Zhou S, Schuetz JD, Bunting KD, Colapietro AM, Sampath J (2001). The ABC transporter Bcrp1/ABCG2 is expressed in a wide variety of stem cells and is a molecular determinant of the side-population phenotype.. NatMed.

[58] Alt R, Wilhelm F, Pelz-Ackermann O, Egger D, Niederwieser D (2009). ABCG2 expression is correlated neither to side population nor to hematopoietic progenitor function in human umbilical cord blood.. Exp Hematol.

