# Supplementary figures and images for: Very Small Embryonic-Like Stem Cells Purified from Umbilical Cord Blood Lack Stem Cell Characteristics
Source: PLoS One. 2012 Apr 3;7(4):e34899. doi: 10.1371/journal.pone.0034899 (PMC3318011; doi:10.1371/journal.pone.0034899)

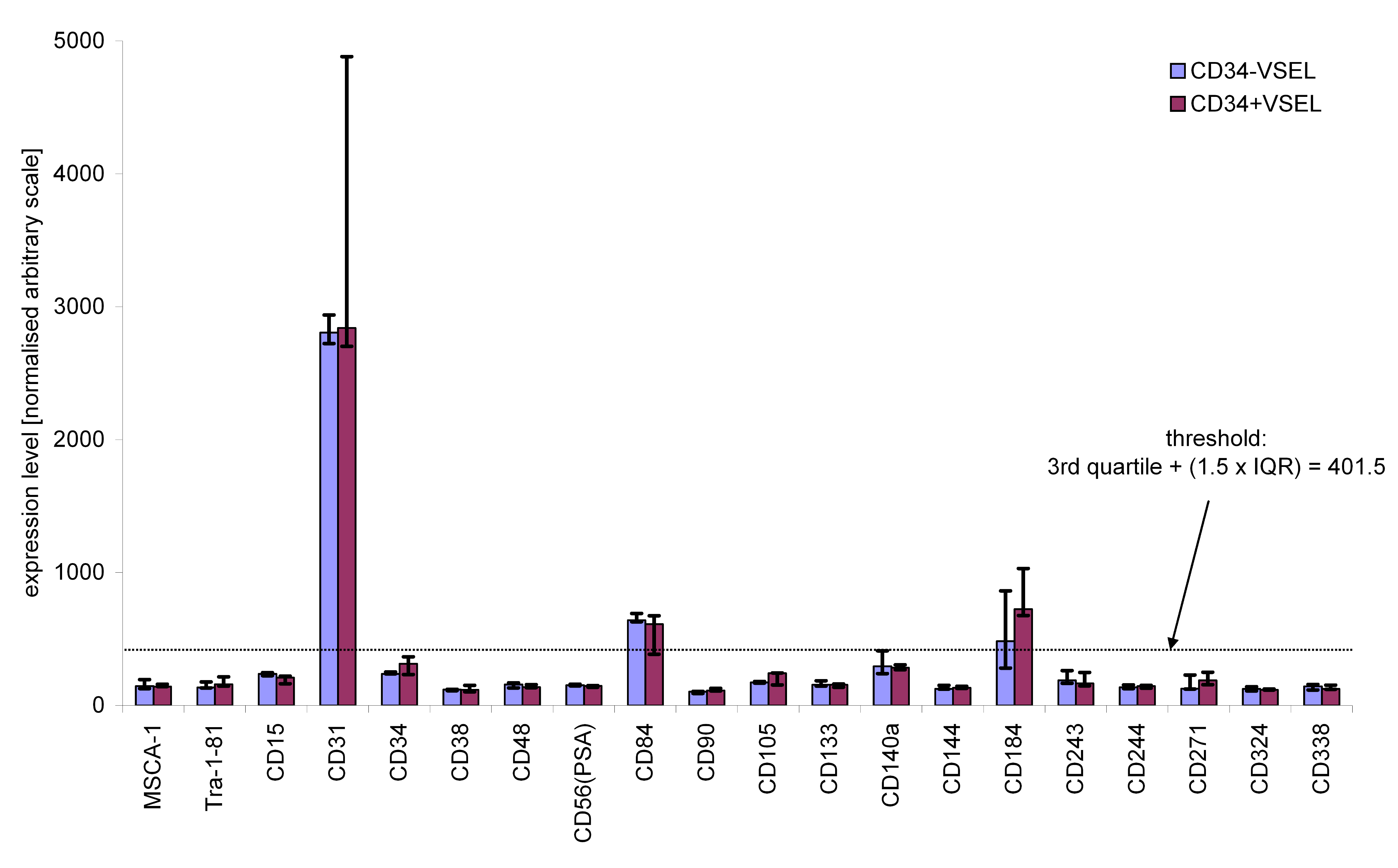

Supplement: Figure S1 — Transcript level of genes coding for selected surface antigens in CD34−VSEL and CD34+VSEL cells. Genes with a median transcript level above the threshold (3rd quartile +1.5× inter quartile range (IQR)) were defined as positive, others as negative. While most transcripts are negative, CD31, CD84 and CD184 (CXCR4) are positive in both cell populations. These data agree with the flow cytometric immunophenotyping of VSEL cells. The level of CD34 mRNA in CD34+VSEL cells was below the set threshold, but was both the highest of all negative values and higher than in CD34−VSEL cell. (TIF) [file pone.0034899.s001.tif]
