# Supplementary material for: Very Small Embryonic-Like Stem Cells Purified from Umbilical Cord Blood Lack Stem Cell Characteristics
Source: PLoS One. 2012 Apr 3;7(4):e34899. doi: 10.1371/journal.pone.0034899 (PMC3318011; doi:10.1371/journal.pone.0034899)
Supplement: Table S1 — Median transcription level of 371 selected pluripotency associated genes queried from www.genecards.org. (DOC) [file pone.0034899.s002.doc]

| **Gene symbol** | **iPSC** | **ESC** | **NSCs** | **MSCs** | **B-cells** | **VSEL** |
| --- | --- | --- | --- | --- | --- | --- |
| ABCG2 | 7,75 | 6,91 | 6,85 | 6,96 | 7,13 | 7,17 |
| ACE | 7,35 | 7,51 | 7,73 | 7,89 | 7,34 | 7,82 |
| ACP1 | 9,81 | 9,75 | 8,94 | 9,35 | 8,36 | 8,66 |
| ACTC1 | 11,04 | 9,02 | 8,33 | 8,04 | 7,70 | 7,58 |
| ACTL6A | 9,01 | 9,49 | 8,69 | 8,83 | 7,40 | 5,67 |
| ACVR1B | 7,07 | 7,24 | 7,37 | 7,15 | 7,54 | 9,25 |
| ACVRL1 | 6,85 | 6,96 | 7,38 | 7,19 | 7,88 | 7,18 |
| ADA | 8,43 | 7,99 | 7,42 | 7,76 | 7,67 | 6,78 |
| ADM | 10,10 | 10,00 | 8,07 | 10,63 | 9,03 | 7,87 |
| AFP | 8,17 | 7,26 | 7,10 | 7,02 | 7,17 | 6,88 |
| AGTR2 | 6,68 | 6,55 | 6,85 | 6,64 | 6,72 | 6,63 |
| AICDA | 6,66 | 6,62 | 6,74 | 6,60 | 6,62 | 6,91 |
| AKT1 | 8,34 | 7,91 | 9,66 | 9,12 | 7,78 | 8,62 |
| AKT2 | 7,21 | 7,09 | 7,26 | 7,03 | 7,79 | 9,20 |
| AKT3 | 7,25 | 7,28 | 7,97 | 7,33 | 6,96 | 8,63 |
| ALB | 6,22 | 6,22 | 6,20 | 6,23 | 6,50 | 6,18 |
| ALPP | 7,22 | 7,40 | 7,45 | 7,44 | 7,67 | 7,41 |
| ANGPT2 | 6,37 | 6,46 | 6,56 | 6,74 | 6,79 | 7,41 |
| APC | 7,20 | 7,19 | 8,42 | 7,31 | 7,19 | 6,26 |
| APOBEC1 | 6,79 | 6,74 | 6,93 | 6,79 | 6,85 | 7,27 |
| AQR | 7,56 | 7,64 | 7,63 | 7,33 | 7,64 | 7,19 |
| ARID1A | 8,36 | 9,05 | 9,24 | 8,36 | 8,93 | 11,29 |
| ARL6IP1 | 10,61 | 11,22 | 11,54 | 9,95 | 9,78 | 8,68 |
| ASH2L | 9,31 | 9,64 | 9,54 | 8,68 | 9,54 | 9,51 |
| ATF2 | 8,04 | 7,98 | 7,79 | 7,48 | 7,19 | 8,43 |
| ATRX | 7,93 | 8,15 | 8,67 | 7,71 | 7,69 | 7,89 |
| AXIN1 | 8,13 | 8,22 | 8,35 | 7,88 | 8,44 | 8,43 |
| BCR | 8,26 | 8,04 | 8,06 | 7,35 | 7,11 | 9,38 |
| BDNF | 7,44 | 7,28 | 7,02 | 7,07 | 6,60 | 7,73 |
| BGLAP | 7,60 | 7,44 | 7,12 | 7,38 | 7,24 | 7,18 |
| BMI1 | 7,86 | 7,86 | 8,46 | 9,25 | 9,96 | 9,62 |
| BMP1 | 7,30 | 7,45 | 7,77 | 8,20 | 8,04 | 7,61 |
| BMP10 | 6,68 | 6,68 | 6,98 | 6,76 | 7,34 | 7,59 |
| BMP15 | 7,53 | 7,51 | 8,40 | 7,90 | 8,16 | 7,74 |
| BMP2 | 7,65 | 8,57 | 7,71 | 7,75 | 7,68 | 7,13 |
| BMP3 | 7,09 | 6,86 | 6,93 | 7,01 | 7,26 | 6,86 |
| BMP4 | 7,89 | 8,06 | 7,28 | 7,19 | 7,16 | 6,20 |
| BMP5 | 6,74 | 6,78 | 6,59 | 8,37 | 6,77 | 6,46 |
| BMP6 | 7,03 | 6,80 | 6,89 | 6,91 | 6,89 | 9,86 |
| BMP7 | 7,65 | 7,72 | 8,30 | 7,24 | 8,09 | 7,43 |
| BMP8A | 7,47 | 7,25 | 7,41 | 7,44 | 7,79 | 7,15 |
| BMPR1A | 9,07 | 9,43 | 8,87 | 8,41 | 7,14 | 6,26 |
| BMPR1B | 6,63 | 6,63 | 7,11 | 6,73 | 7,91 | 6,61 |
| BMPR2 | 7,31 | 7,32 | 7,44 | 7,27 | 7,06 | 6,05 |
| BNIP3 | 8,81 | 10,67 | 9,02 | 10,62 | 7,23 | 8,96 |
| BNIP3L | 8,94 | 9,82 | 10,18 | 10,36 | 8,88 | 12,33 |
| BRCA1 | 7,93 | 8,05 | 7,72 | 7,48 | 7,11 | 7,15 |
| C3orf63 | 8,06 | 8,42 | 8,13 | 8,45 | 10,02 | 7,49 |
| CASP6 | 8,55 | 8,31 | 9,01 | 8,22 | 9,01 | 8,73 |
| CBX5 | 8,35 | 9,48 | 9,50 | 7,64 | 6,66 | 7,08 |
| CCK | 7,00 | 7,02 | 6,95 | 7,24 | 7,43 | 8,54 |
| CCNF | 8,15 | 8,04 | 7,39 | 7,33 | 7,49 | 7,80 |
| CD36 | 7,10 | 6,63 | 6,69 | 7,34 | 6,64 | 7,98 |
| CDC34 | 9,13 | 8,98 | 8,89 | 8,23 | 7,51 | 7,74 |
| CDCA8 | 9,06 | 8,90 | 8,84 | 8,06 | 7,49 | 7,63 |
| CDH1 | 9,31 | 9,49 | 6,90 | 6,76 | 6,93 | 7,83 |
| CDH5 | 6,89 | 6,96 | 7,04 | 6,89 | 6,94 | 7,13 |
| CDKN1A | 8,20 | 7,60 | 8,58 | 8,96 | 7,36 | 10,22 |
| CDKN1B | 8,53 | 9,01 | 9,79 | 8,51 | 10,39 | 9,43 |
| CDKN2A | 7,07 | 6,97 | 6,88 | 7,66 | 8,48 | 6,45 |
| CDX2 | 6,95 | 6,94 | 6,80 | 6,92 | 7,75 | 8,69 |
| CHD1 | 8,41 | 8,98 | 8,41 | 8,33 | 8,30 | 7,69 |
| CLDN3 | 7,84 | 7,77 | 6,84 | 7,17 | 7,41 | 6,51 |
| CSF1 | 7,29 | 7,30 | 7,53 | 7,57 | 7,68 | 8,78 |
| CSF1R | 7,20 | 7,12 | 7,30 | 7,49 | 7,73 | 8,09 |
| CSF2 | 7,19 | 7,20 | 7,49 | 7,45 | 7,34 | 6,38 |
| CSF3 | 7,21 | 7,21 | 7,13 | 7,17 | 7,93 | 7,69 |
| CTCF | 9,31 | 9,84 | 9,33 | 8,66 | 9,67 | 8,78 |
| CTCFL | 8,38 | 8,31 | 8,37 | 7,56 | 7,54 | 8,57 |
| CTNNB1 | 8,57 | 7,96 | 7,58 | 8,68 | 7,75 | 9,86 |
| DAZL | 7,66 | 7,92 | 7,22 | 7,43 | 7,29 | 7,07 |
| DES | 7,50 | 7,34 | 7,18 | 7,52 | 7,87 | 7,94 |
| DLX5 | 6,82 | 7,66 | 9,86 | 7,38 | 6,94 | 6,80 |
| DMRT1 | 6,59 | 6,54 | 7,13 | 6,58 | 6,96 | 6,88 |
| DNMT1 | 9,89 | 10,18 | 9,37 | 9,30 | 8,91 | 10,01 |
| DNMT3A | 8,72 | 8,14 | 7,91 | 7,59 | 7,48 | 7,81 |
| DNMT3B | 11,85 | 11,64 | 7,88 | 7,36 | 7,26 | 8,52 |
| DPPA4 | 10,48 | 10,05 | 6,52 | 6,51 | 7,16 | 8,11 |
| DSP | 9,72 | 9,63 | 7,17 | 7,46 | 8,64 | 7,27 |
| DUSP6 | 9,44 | 8,29 | 10,30 | 9,68 | 7,72 | 7,69 |
| DVL1 | 8,43 | 8,65 | 8,00 | 8,85 | 7,95 | 7,06 |
| DYRK1A | 7,08 | 7,33 | 7,28 | 7,20 | 7,64 | 11,38 |
| EED | 7,85 | 7,88 | 7,31 | 7,79 | 7,97 | 6,49 |
| EGR1 | 8,55 | 9,22 | 7,96 | 10,06 | 8,06 | 7,95 |
| ENO2 | 8,52 | 8,82 | 9,14 | 7,84 | 7,55 | 8,65 |
| EPAS1 | 7,24 | 7,14 | 7,29 | 7,72 | 6,05 | 6,38 |
| EPO | 6,97 | 6,88 | 6,75 | 6,85 | 7,22 | 7,34 |
| EPX | 6,88 | 7,00 | 7,19 | 6,96 | 7,61 | 7,18 |
| ESRRB | 6,90 | 7,04 | 7,13 | 6,98 | 7,04 | 7,29 |
| EZH1 | 7,53 | 7,82 | 8,14 | 7,86 | 8,65 | 9,34 |
| EZH2 | 8,49 | 9,02 | 9,14 | 7,77 | 7,07 | 9,89 |
| FGF2 | 8,55 | 8,71 | 6,89 | 7,78 | 7,15 | 7,60 |
| FGF4 | 7,55 | 7,49 | 7,26 | 7,28 | 6,91 | 7,21 |
| FGFR1 | 8,06 | 8,75 | 8,62 | 8,49 | 8,63 | 7,88 |
| FGFR2 | 7,45 | 7,06 | 7,01 | 6,82 | 7,11 | 7,12 |
| FGFR3 | 7,50 | 8,25 | 7,79 | 6,96 | 6,92 | 8,61 |
| FGFR4 | 7,66 | 7,52 | 7,05 | 7,09 | 7,15 | 7,73 |
| FLT3LG | 6,73 | 6,77 | 6,85 | 7,10 | 6,97 | 6,92 |
| FOS | 8,45 | 9,01 | 7,93 | 9,90 | 7,15 | 8,84 |
| FOXA1 | 6,47 | 6,72 | 6,41 | 6,55 | 6,61 | 6,29 |
| FOXA2 | 6,82 | 7,26 | 6,83 | 6,83 | 7,36 | 6,92 |
| FOXD3 | 7,21 | 7,40 | 6,69 | 6,69 | 6,54 | 6,88 |
| FRZB | 7,02 | 8,71 | 7,00 | 7,55 | 7,08 | 6,55 |
| FZD1 | 7,00 | 7,47 | 8,05 | 8,38 | 7,09 | 6,83 |
| FZD10 | 6,80 | 7,44 | 6,84 | 6,67 | 6,89 | 7,40 |
| FZD2 | 7,85 | 10,06 | 9,02 | 7,63 | 6,89 | 7,10 |
| FZD3 | 7,80 | 8,09 | 10,00 | 6,69 | 6,93 | 7,10 |
| FZD4 | 7,49 | 7,61 | 7,66 | 7,86 | 7,17 | 8,22 |
| FZD5 | 8,42 | 9,26 | 8,75 | 7,20 | 6,88 | 8,13 |
| FZD6 | 7,95 | 7,84 | 7,88 | 7,88 | 6,81 | 7,04 |
| FZD7 | 9,98 | 10,58 | 8,46 | 8,15 | 6,77 | 7,31 |
| FZD8 | 6,92 | 7,84 | 7,42 | 6,85 | 7,05 | 6,55 |
| FZD9 | 7,29 | 7,40 | 7,22 | 7,07 | 7,57 | 7,98 |
| GAB1 | 7,23 | 7,25 | 8,65 | 7,27 | 7,16 | 9,61 |
| GAD1 | 6,75 | 7,22 | 8,98 | 6,58 | 7,06 | 6,50 |
| GAD2 | 6,99 | 6,80 | 6,87 | 6,92 | 7,32 | 7,10 |
| GADD45A | 9,15 | 8,29 | 8,49 | 9,65 | 8,11 | 10,45 |
| GATA4 | 7,16 | 7,13 | 7,74 | 7,31 | 8,35 | 8,19 |
| GATA6 | 6,61 | 6,90 | 6,52 | 6,64 | 6,62 | 6,34 |
| GBX2 | 6,70 | 6,77 | 6,97 | 6,61 | 6,88 | 7,18 |
| GFAP | 6,99 | 6,92 | 7,59 | 6,99 | 6,39 | 7,69 |
| GHR | 6,55 | 6,99 | 6,26 | 9,28 | 6,37 | 6,91 |
| GJA1 | 11,89 | 12,22 | 11,76 | 11,50 | 6,23 | 10,74 |
| GLI1 | 7,63 | 7,44 | 7,19 | 7,12 | 7,86 | 7,46 |
| GNAS | 9,60 | 9,85 | 10,68 | 9,64 | 10,24 | 11,39 |
| GNL3 | 10,22 | 10,30 | 8,65 | 9,54 | 9,01 | 8,59 |
| GPR182 | 6,88 | 6,98 | 7,28 | 7,00 | 7,60 | 7,28 |
| GRB2 | 8,86 | 9,01 | 9,07 | 8,90 | 9,81 | 8,63 |
| GSK3A | 8,56 | 8,69 | 9,33 | 8,81 | 9,06 | 7,87 |
| GSK3B | 7,85 | 7,60 | 8,70 | 7,81 | 7,26 | 7,68 |
| H3F3A | 12,54 | 12,57 | 13,16 | 11,29 | 12,24 | 13,68 |
| H3F3B | 11,36 | 11,86 | 11,93 | 10,74 | 11,23 | 13,28 |
| HBD | 6,60 | 6,60 | 6,73 | 6,84 | 8,65 | 8,81 |
| HCFC1 | 7,63 | 7,73 | 7,72 | 7,61 | 8,27 | 8,81 |
| HDAC1 | 10,10 | 9,91 | 9,07 | 9,21 | 10,25 | 9,42 |
| HDAC2 | 8,83 | 9,19 | 8,81 | 8,38 | 7,44 | 8,85 |
| HDAC3 | 9,18 | 9,35 | 8,95 | 8,93 | 8,96 | 7,60 |
| HDAC9 | 7,05 | 7,14 | 7,19 | 6,97 | 9,22 | 8,19 |
| HGF | 6,39 | 6,33 | 6,60 | 8,52 | 6,71 | 6,86 |
| HIF1A | 11,05 | 11,47 | 10,80 | 12,09 | 10,70 | 10,77 |
| HMGA2 | 6,92 | 6,97 | 7,03 | 7,43 | 6,07 | 7,20 |
| HMOX1 | 8,17 | 7,63 | 7,58 | 9,47 | 7,26 | 7,53 |
| HNF1A | 7,38 | 7,30 | 7,27 | 7,42 | 7,23 | 7,62 |
| HOXA1 | 6,46 | 7,00 | 6,63 | 7,45 | 6,78 | 6,84 |
| HOXB1 | 6,97 | 7,37 | 7,22 | 7,31 | 7,58 | 7,81 |
| HPRT1 | 8,14 | 8,20 | 7,64 | 8,41 | 8,07 | 4,32 |
| HRAS | 8,34 | 7,95 | 8,71 | 8,54 | 7,14 | 8,22 |
| HSPB2 | 7,03 | 7,07 | 7,25 | 7,82 | 7,30 | 7,64 |
| IAPP | 7,56 | 7,53 | 7,28 | 7,51 | 6,65 | 7,03 |
| IBSP | 6,76 | 6,84 | 6,79 | 6,86 | 7,55 | 6,96 |
| IFNG | 7,04 | 6,92 | 6,94 | 6,86 | 6,95 | 6,92 |
| IGF1 | 6,85 | 6,82 | 6,99 | 6,80 | 7,22 | 8,15 |
| IGF2 | 7,14 | 7,25 | 7,20 | 7,40 | 6,98 | 5,97 |
| IL11 | 7,36 | 7,04 | 6,99 | 7,17 | 7,33 | 7,93 |
| IL11RA | 7,21 | 7,31 | 7,61 | 7,82 | 7,40 | 7,96 |
| IL1A | 7,07 | 6,84 | 6,92 | 6,69 | 6,96 | 6,47 |
| IL2RG | 7,11 | 7,24 | 7,58 | 7,33 | 8,77 | 7,85 |
| IL3 | 6,39 | 6,36 | 6,47 | 6,41 | 6,53 | 6,92 |
| IL6R | 7,06 | 6,69 | 6,55 | 6,98 | 8,27 | 7,71 |
| IL6ST | 6,72 | 6,82 | 8,04 | 7,54 | 6,98 | 12,42 |
| IL7 | 6,15 | 6,10 | 6,31 | 7,87 | 7,01 | 6,67 |
| ITGAL | 6,92 | 7,07 | 6,95 | 6,91 | 8,00 | 7,48 |
| JAK1 | 8,27 | 7,86 | 8,84 | 8,47 | 9,63 | 10,21 |
| JAK2 | 6,83 | 6,74 | 6,95 | 7,70 | 7,45 | 10,33 |
| JAK3 | 7,31 | 7,12 | 6,97 | 7,19 | 7,19 | 8,44 |
| JARID2 | 10,39 | 10,09 | 8,15 | 7,72 | 9,34 | 11,03 |
| JUN | 7,98 | 7,64 | 8,99 | 8,96 | 7,83 | 10,42 |
| KATNA1 | 8,69 | 8,52 | 7,97 | 8,38 | 8,50 | 7,67 |
| KATNB1 | 7,87 | 7,64 | 7,39 | 7,31 | 6,86 | 8,16 |
| KDM5B | 9,36 | 10,07 | 9,44 | 8,50 | 7,75 | 8,45 |
| KIT | 8,54 | 8,94 | 6,77 | 6,77 | 7,17 | 9,04 |
| KITLG | 6,84 | 6,69 | 7,83 | 7,49 | 6,56 | 6,87 |
| KLF2 | 7,25 | 7,20 | 7,30 | 7,42 | 10,33 | 9,47 |
| KLF4 | 7,19 | 7,65 | 6,74 | 7,89 | 7,25 | 8,25 |
| KLF5 | 7,52 | 7,28 | 6,75 | 6,97 | 6,86 | 7,98 |
| KLK2 | 7,05 | 6,97 | 7,22 | 7,00 | 7,70 | 7,58 |
| KRAS | 7,35 | 7,61 | 9,00 | 7,76 | 7,80 | 8,73 |
| KRT19 | 8,20 | 8,23 | 6,65 | 6,80 | 6,21 | 6,09 |
| LEF1 | 7,10 | 7,34 | 7,15 | 7,37 | 7,34 | 6,75 |
| LEFTY1 | 9,78 | 8,20 | 6,84 | 6,68 | 6,92 | 8,77 |
| LIF | 7,63 | 7,15 | 7,03 | 7,77 | 7,43 | 7,16 |
| LIFR | 6,81 | 6,99 | 8,97 | 7,62 | 7,06 | 6,50 |
| LIN28A | 12,04 | 12,34 | 6,90 | 6,75 | 7,12 | 6,99 |
| MAGEA4 | 7,17 | 6,51 | 6,55 | 6,49 | 6,45 | 6,50 |
| MAP2K2 | 8,59 | 7,99 | 8,72 | 8,25 | 7,57 | 7,48 |
| MAP2K3 | 8,21 | 7,80 | 7,65 | 8,55 | 8,41 | 7,82 |
| MAPK1 | 8,31 | 8,11 | 8,95 | 7,98 | 7,44 | 8,15 |
| MAPK14 | 8,04 | 7,93 | 7,86 | 7,77 | 7,60 | 9,90 |
| MAPK3 | 8,15 | 8,20 | 8,78 | 8,83 | 8,82 | 7,25 |
| MECOM | 6,67 | 6,68 | 6,79 | 6,82 | 6,72 | 6,64 |
| MEF2A | 7,84 | 7,76 | 7,32 | 7,72 | 7,80 | 8,35 |
| MLANA | 6,85 | 6,87 | 7,11 | 6,88 | 6,82 | 7,64 |
| MRAS | 7,31 | 7,32 | 8,10 | 8,17 | 7,36 | 8,73 |
| MSTN | 5,98 | 5,98 | 6,02 | 6,14 | 5,97 | 6,51 |
| MSX2 | 6,96 | 7,41 | 6,91 | 7,00 | 6,80 | 6,46 |
| MUC1 | 7,05 | 7,23 | 7,22 | 7,16 | 7,51 | 7,63 |
| MYC | 8,33 | 8,40 | 8,12 | 8,90 | 8,75 | 8,19 |
| MYCN | 7,46 | 7,31 | 6,92 | 6,94 | 7,85 | 8,16 |
| MYH11 | 7,11 | 7,03 | 7,06 | 7,08 | 7,08 | 7,35 |
| MYH6 | 6,62 | 6,62 | 6,43 | 6,59 | 6,66 | 7,48 |
| MYOD1 | 7,21 | 7,35 | 7,55 | 7,41 | 7,66 | 7,81 |
| NANOG | 10,89 | 9,82 | 6,62 | 6,87 | 7,05 | 7,53 |
| NCAM1 | 6,78 | 6,81 | 7,26 | 6,69 | 7,25 | 7,53 |
| NES | 8,66 | 8,61 | 9,69 | 7,84 | 7,72 | 6,96 |
| NFKB1 | 7,55 | 7,48 | 8,03 | 8,23 | 9,45 | 6,55 |
| NGF | 7,05 | 7,05 | 6,77 | 6,93 | 7,57 | 8,24 |
| NIN | 7,13 | 7,17 | 7,16 | 7,64 | 7,49 | 7,69 |
| NKX2-5 | 7,72 | 7,62 | 7,64 | 7,80 | 7,35 | 7,75 |
| NME4 | 10,11 | 9,45 | 9,96 | 9,67 | 8,49 | 7,78 |
| NOTCH2 | 8,26 | 8,51 | 9,10 | 9,22 | 9,10 | 9,98 |
| NOTCH4 | 7,33 | 7,39 | 7,51 | 7,44 | 7,55 | 7,78 |
| NR0B1 | 6,56 | 6,54 | 6,77 | 6,79 | 6,85 | 6,92 |
| NR1H2 | 7,68 | 7,66 | 7,68 | 7,90 | 7,55 | 7,95 |
| NR2F1 | 6,75 | 7,50 | 8,05 | 9,26 | 6,80 | 8,33 |
| NR2F2 | 6,47 | 7,03 | 7,50 | 8,70 | 6,52 | 5,30 |
| NR2F6 | 8,83 | 8,59 | 7,95 | 8,07 | 7,37 | 7,54 |
| NR5A1 | 6,80 | 6,87 | 6,70 | 6,76 | 7,22 | 7,43 |
| NR5A2 | 7,36 | 6,65 | 6,45 | 6,41 | 6,44 | 6,54 |
| NR6A1 | 7,36 | 7,56 | 7,57 | 7,66 | 8,09 | 8,04 |
| NRAS | 9,39 | 9,48 | 9,15 | 8,73 | 7,57 | 8,55 |
| NRIP1 | 7,23 | 8,06 | 8,73 | 8,35 | 8,47 | 9,06 |
| NTF3 | 7,26 | 7,42 | 7,30 | 7,16 | 7,42 | 10,02 |
| NTRK1 | 6,75 | 6,54 | 6,38 | 6,42 | 7,02 | 7,85 |
| NTRK2 | 6,81 | 6,94 | 7,06 | 6,87 | 7,61 | 7,12 |
| NTRK3 | 7,04 | 7,09 | 7,46 | 7,10 | 7,94 | 8,45 |
| OSM | 6,93 | 6,95 | 6,69 | 6,92 | 7,33 | 7,50 |
| PAK4 | 8,28 | 8,76 | 8,62 | 8,15 | 8,55 | 8,61 |
| PARP1 | 10,58 | 10,77 | 10,58 | 9,04 | 10,48 | 9,40 |
| PCSK6 | 6,85 | 6,91 | 6,92 | 6,87 | 7,31 | 8,25 |
| PDGFA | 9,06 | 7,88 | 7,90 | 7,73 | 7,32 | 11,56 |
| PDGFB | 7,41 | 7,06 | 7,35 | 7,09 | 7,29 | 8,48 |
| PDGFC | 7,90 | 7,24 | 8,68 | 8,90 | 6,19 | 10,56 |
| PDGFD | 7,27 | 7,35 | 7,63 | 7,09 | 6,74 | 7,08 |
| PDGFRA | 6,97 | 7,16 | 7,12 | 7,55 | 6,59 | 8,11 |
| PDHB | 10,51 | 10,18 | 9,90 | 9,66 | 9,10 | 8,28 |
| PDPK1 | 7,22 | 7,69 | 7,65 | 7,44 | 8,10 | 8,42 |
| PDX1 | 7,31 | 7,20 | 7,18 | 6,94 | 7,58 | 7,54 |
| PECAM1 | 7,19 | 7,17 | 7,77 | 7,33 | 8,72 | 11,33 |
| PHB | 9,66 | 9,40 | 8,54 | 8,66 | 8,19 | 7,94 |
| PHC1 | 10,40 | 10,12 | 8,82 | 7,82 | 8,56 | 8,38 |
| PHC3 | 6,99 | 7,16 | 6,91 | 7,22 | 7,19 | 7,86 |
| PIGA | 7,98 | 7,89 | 7,35 | 7,74 | 7,13 | 9,06 |
| PIK3C2A | 7,56 | 7,91 | 7,53 | 7,61 | 6,66 | 7,84 |
| PIK3C2B | 8,22 | 8,68 | 8,67 | 7,92 | 9,87 | 7,60 |
| PIK3C2G | 6,64 | 6,62 | 6,81 | 6,75 | 6,78 | 6,39 |
| PIK3C3 | 7,21 | 7,09 | 7,43 | 7,09 | 7,53 | 6,65 |
| PIK3CA | 7,03 | 6,98 | 7,06 | 7,52 | 7,19 | 7,96 |
| PIK3CB | 7,70 | 7,94 | 7,23 | 7,40 | 7,03 | 9,39 |
| PIK3CD | 7,71 | 7,53 | 7,00 | 7,11 | 9,40 | 7,97 |
| PIK3CG | 6,43 | 6,42 | 6,43 | 6,44 | 6,96 | 7,25 |
| PIK3R1 | 7,09 | 7,53 | 8,43 | 7,75 | 7,90 | 9,08 |
| PIK3R2 | 7,55 | 7,63 | 8,33 | 7,55 | 8,65 | 8,15 |
| PIK3R3 | 7,04 | 7,31 | 8,40 | 7,10 | 6,51 | 7,86 |
| PIK3R4 | 7,45 | 7,53 | 7,00 | 7,56 | 7,28 | 6,12 |
| PIK3R5 | 7,31 | 7,38 | 7,00 | 7,14 | 7,55 | 7,60 |
| PODXL | 11,57 | 11,50 | 8,59 | 6,54 | 7,06 | 8,07 |
| POSTN | 6,86 | 6,66 | 6,78 | 7,61 | 6,92 | 6,36 |
| POU5F1 | 11,91 | 11,33 | 8,52 | 8,15 | 9,06 | 8,92 |
| PPP1R8 | 9,63 | 9,50 | 9,74 | 9,18 | 9,01 | 8,34 |
| PRKG1 | 6,93 | 7,10 | 7,05 | 6,75 | 6,83 | 7,02 |
| PRMT7 | 8,17 | 7,98 | 7,14 | 7,87 | 7,34 | 7,00 |
| PROM1 | 9,81 | 9,35 | 9,07 | 6,44 | 6,74 | 7,24 |
| PTHLH | 6,72 | 6,53 | 6,59 | 6,44 | 6,66 | 6,99 |
| PTN | 7,43 | 7,59 | 7,72 | 7,36 | 7,47 | 7,68 |
| PTPN11 | 7,20 | 7,06 | 7,14 | 6,98 | 7,39 | 8,32 |
| RAC1 | 10,55 | 10,72 | 11,21 | 10,48 | 9,93 | 9,56 |
| RAC2 | 7,42 | 7,31 | 7,44 | 8,45 | 10,39 | 8,48 |
| RAC3 | 8,99 | 7,96 | 7,94 | 7,26 | 6,90 | 7,58 |
| RAF1 | 8,47 | 8,46 | 8,83 | 8,55 | 8,80 | 8,71 |
| RARA | 7,36 | 7,37 | 7,38 | 7,24 | 7,65 | 9,16 |
| RB1 | 7,63 | 7,75 | 8,63 | 7,89 | 8,63 | 8,72 |
| RCVRN | 7,22 | 7,17 | 7,58 | 7,32 | 7,22 | 7,23 |
| REST | 7,88 | 8,41 | 8,18 | 8,05 | 8,53 | 9,87 |
| RHO | 7,20 | 7,28 | 7,39 | 7,27 | 7,76 | 8,09 |
| RIF1 | 7,78 | 7,72 | 7,60 | 7,21 | 6,69 | 7,33 |
| RLBP1 | 7,02 | 6,93 | 7,05 | 7,00 | 7,16 | 6,60 |
| RPE65 | 6,03 | 6,03 | 7,41 | 6,10 | 6,29 | 6,07 |
| RRAS | 8,14 | 7,81 | 7,81 | 9,31 | 7,59 | 7,93 |
| RRAS2 | 10,51 | 10,00 | 8,32 | 9,80 | 8,77 | 6,50 |
| RUNX1 | 6,98 | 6,97 | 6,89 | 7,27 | 7,29 | 8,30 |
| RUNX2 | 6,75 | 6,82 | 6,91 | 7,21 | 7,19 | 7,13 |
| RXRB | 7,20 | 7,07 | 7,50 | 7,18 | 7,38 | 8,20 |
| S100A1 | 7,18 | 7,24 | 7,07 | 7,28 | 7,40 | 10,29 |
| S1PR1 | 6,83 | 6,61 | 7,50 | 7,77 | 9,03 | 7,40 |
| S1PR2 | 7,25 | 7,33 | 6,95 | 7,16 | 7,34 | 8,11 |
| S1PR4 | 7,63 | 7,68 | 7,84 | 7,98 | 10,47 | 8,31 |
| S1PR5 | 7,32 | 7,11 | 6,96 | 7,18 | 6,98 | 6,74 |
| SALL1 | 8,24 | 9,80 | 10,07 | 6,88 | 6,52 | 6,86 |
| SH3GLB1 | 8,85 | 8,62 | 8,95 | 9,87 | 8,74 | 9,32 |
| SHC1 | 9,80 | 9,03 | 8,88 | 10,30 | 8,41 | 9,38 |
| SLC2A1 | 8,33 | 8,60 | 7,83 | 7,99 | 8,27 | 7,53 |
| SMAD1 | 8,47 | 8,44 | 9,17 | 7,99 | 7,43 | 7,80 |
| SMAD2 | 8,34 | 8,69 | 7,97 | 8,44 | 8,40 | 9,22 |
| SMAD3 | 7,67 | 7,58 | 7,92 | 8,31 | 8,49 | 8,80 |
| SMAD4 | 7,02 | 7,42 | 7,09 | 6,93 | 6,70 | 7,85 |
| SMAD5 | 7,96 | 8,32 | 8,51 | 8,43 | 7,33 | 6,68 |
| SMAD6 | 7,28 | 7,11 | 6,84 | 7,39 | 7,18 | 6,73 |
| SMAD7 | 8,06 | 8,02 | 6,69 | 8,01 | 6,70 | 8,29 |
| SMAD9 | 6,87 | 6,86 | 7,26 | 7,31 | 7,85 | 8,81 |
| SMARCA2 | 7,41 | 7,22 | 8,13 | 7,74 | 7,53 | 8,40 |
| SMARCB1 | 7,87 | 7,99 | 8,00 | 7,86 | 8,32 | 7,82 |
| SMARCC1 | 9,15 | 9,40 | 8,94 | 8,02 | 7,95 | 8,05 |
| SMARCC2 | 7,99 | 7,96 | 8,80 | 8,11 | 7,86 | 8,80 |
| SMARCE1 | 9,46 | 10,39 | 9,55 | 9,46 | 9,85 | 7,11 |
| SMO | 8,08 | 8,02 | 8,42 | 7,57 | 7,95 | 7,23 |
| SOD2 | 8,00 | 7,92 | 7,61 | 8,38 | 7,37 | 10,22 |
| SOS1 | 7,34 | 7,56 | 7,95 | 7,72 | 8,00 | 9,46 |
| SOS2 | 7,15 | 7,20 | 7,70 | 7,13 | 7,40 | 6,99 |
| SOX17 | 6,67 | 7,08 | 6,72 | 6,95 | 6,57 | 7,52 |
| SOX2 | 9,43 | 10,23 | 9,45 | 6,77 | 7,96 | 6,54 |
| SOX9 | 7,06 | 8,04 | 11,23 | 7,10 | 6,84 | 8,05 |
| SPHK1 | 7,54 | 7,35 | 7,48 | 8,50 | 7,37 | 8,94 |
| SPP1 | 9,64 | 7,64 | 8,38 | 6,97 | 5,80 | 4,81 |
| STAT3 | 8,78 | 8,59 | 9,35 | 9,00 | 8,06 | 11,61 |
| SUZ12 | 7,46 | 7,57 | 7,63 | 7,39 | 7,97 | 6,94 |
| TAGLN | 11,26 | 8,57 | 7,81 | 10,90 | 7,52 | 7,28 |
| TAP1 | 7,99 | 7,53 | 7,87 | 8,64 | 9,99 | 9,32 |
| TAPBP | 8,30 | 7,92 | 8,58 | 8,27 | 9,31 | 8,89 |
| TCF3 | 8,74 | 9,26 | 9,03 | 8,08 | 9,85 | 8,47 |
| TCF4 | 8,33 | 8,94 | 10,55 | 7,70 | 9,45 | 9,33 |
| TCF7 | 8,48 | 8,74 | 8,91 | 9,03 | 10,74 | 9,90 |
| TCF7L1 | 9,54 | 9,73 | 8,90 | 7,67 | 7,47 | 7,95 |
| TCF7L2 | 8,46 | 9,52 | 8,15 | 8,10 | 7,37 | 7,28 |
| TCL1A | 7,60 | 7,17 | 7,43 | 7,16 | 11,58 | 7,49 |
| TDGF1 | 11,86 | 11,27 | 6,91 | 6,89 | 7,11 | 6,40 |
| TDRD12 | 7,55 | 7,37 | 7,00 | 6,83 | 6,77 | 7,16 |
| TDRD7 | 7,20 | 7,40 | 7,84 | 7,69 | 8,20 | 7,33 |
| TERT | 7,34 | 7,34 | 7,19 | 6,87 | 7,43 | 7,71 |
| TFAP2A | 6,79 | 7,14 | 7,30 | 7,33 | 7,57 | 7,94 |
| TFAP2C | 8,32 | 8,14 | 7,17 | 7,13 | 6,73 | 6,51 |
| TGFB1 | 7,39 | 7,15 | 7,15 | 8,01 | 7,44 | 9,60 |
| TGFB2 | 6,84 | 6,72 | 7,95 | 7,26 | 7,11 | 7,13 |
| TGFB3 | 6,85 | 6,88 | 7,19 | 7,26 | 7,68 | 7,67 |
| TGFBR1 | 7,99 | 8,62 | 8,12 | 8,04 | 7,59 | 8,05 |
| TGFBR2 | 7,50 | 7,24 | 6,96 | 9,01 | 9,26 | 7,86 |
| THAP11 | 8,03 | 7,88 | 8,07 | 7,82 | 8,84 | 8,52 |
| THAP7 | 7,78 | 7,77 | 7,89 | 7,85 | 7,72 | 7,99 |
| THPO | 6,67 | 6,64 | 6,74 | 6,74 | 6,77 | 6,75 |
| TLE1 | 8,55 | 8,43 | 9,06 | 7,55 | 8,13 | 6,52 |
| TMSB4X | 12,40 | 12,69 | 12,89 | 12,73 | 12,29 | 13,47 |
| TNFAIP6 | 7,21 | 6,84 | 6,83 | 9,44 | 6,94 | 7,85 |
| TP53 | 9,33 | 8,58 | 8,21 | 7,81 | 7,36 | 8,96 |
| TPBG | 9,29 | 9,60 | 8,26 | 9,62 | 7,02 | 7,89 |
| TSC22D3 | 7,59 | 7,89 | 7,35 | 7,93 | 9,52 | 9,08 |
| TTR | 7,28 | 7,44 | 7,46 | 7,32 | 7,51 | 6,85 |
| TUBB3 | 11,86 | 11,35 | 12,70 | 10,65 | 7,69 | 9,47 |
| TYK2 | 7,99 | 7,94 | 8,25 | 8,56 | 9,21 | 7,76 |
| TYR | 6,89 | 6,76 | 6,90 | 6,69 | 6,82 | 6,98 |
| UBE2B | 7,46 | 7,67 | 7,63 | 8,10 | 7,89 | 7,94 |
| UBE2D2 | 9,89 | 9,96 | 9,25 | 9,33 | 9,75 | 8,84 |
| UBE2K | 9,59 | 9,67 | 8,63 | 8,71 | 8,27 | 9,53 |
| UTF1 | 7,44 | 7,28 | 6,74 | 6,89 | 6,89 | 6,91 |
| WNT1 | 6,68 | 6,77 | 6,69 | 6,70 | 6,79 | 6,79 |
| WNT10B | 7,65 | 7,56 | 8,07 | 7,63 | 8,62 | 6,75 |
| WNT11 | 6,91 | 7,20 | 7,62 | 7,43 | 8,94 | 11,95 |
| WNT16 | 7,35 | 7,29 | 7,26 | 7,47 | 7,97 | 7,20 |
| WNT2 | 6,96 | 6,92 | 7,37 | 6,99 | 7,57 | 7,20 |
| WNT2B | 6,85 | 6,92 | 6,79 | 7,04 | 7,01 | 7,55 |
| WNT3 | 7,28 | 7,10 | 7,01 | 6,92 | 7,83 | 6,77 |
| WNT4 | 6,81 | 6,84 | 6,86 | 6,84 | 7,05 | 7,91 |
| WNT5A | 6,94 | 7,15 | 6,97 | 9,21 | 6,77 | 7,71 |
| WNT5B | 7,22 | 7,10 | 7,06 | 7,94 | 7,42 | 8,06 |
| WNT6 | 7,14 | 7,24 | 7,20 | 7,31 | 7,34 | 7,94 |
| WNT7A | 7,08 | 7,11 | 7,15 | 7,16 | 7,32 | 7,61 |
| WNT8B | 6,92 | 7,05 | 6,68 | 6,84 | 7,17 | 7,53 |
| WRN | 8,11 | 8,16 | 7,44 | 7,95 | 7,95 | 6,75 |
| WWP2 | 7,50 | 7,49 | 7,72 | 7,49 | 8,57 | 7,38 |
| YBX1 | 12,33 | 12,07 | 12,10 | 11,27 | 10,03 | 9,71 |
| ZCCHC11 | 7,98 | 8,28 | 7,76 | 7,98 | 7,88 | 7,94 |
| ZEB2 | 6,71 | 7,09 | 7,67 | 7,73 | 6,92 | 11,52 |
| ZIC3 | 9,59 | 10,35 | 8,83 | 7,53 | 7,50 | 7,46 |
