# Supplementary material for: Very Small Embryonic-Like Stem Cells Purified from Umbilical Cord Blood Lack Stem Cell Characteristics
Source: PLoS One. 2012 Apr 3;7(4):e34899. doi: 10.1371/journal.pone.0034899 (PMC3318011; doi:10.1371/journal.pone.0034899)
Supplement: Table S2 — Transcription data sets used in the microarray analysis. (DOC) [file pone.0034899.s003.doc]

| **Cell population** | **GEO series** | **Samples** | **Description** | **Reference** |
| --- | --- | --- | --- | --- |
| B-cells | [GSE6691](http://www.ncbi.nlm.nih.gov/geo/query/acc.cgi?acc=GSE6691) | GSM154423-30 | FACS sorted CD19+ B-lymphocyted from peripheral blood |  |
| dendritic cells | [GSE11943](http://www.ncbi.nlm.nih.gov/geo/query/acc.cgi?acc=GSE11943) | GSM301723-26 | FACS sorted CD1c+ cells from peripheral blood | n/a |
| erythroid cells | [GSE24598](http://www.ncbi.nlm.nih.gov/geo/query/acc.cgi?acc=GSE24598) | GSM250022-24 | MACS sorted CD235a+ cells from bone marrow |  |
| ESCs | [GSE22499](http://www.ncbi.nlm.nih.gov/geo/query/acc.cgi?acc=GSE22499) | GSM574066-71 | embryonic stem cell lines BG03, H9 and WIBR1 |  |
| HMVECs | [GSE18113](http://www.ncbi.nlm.nih.gov/geo/query/acc.cgi?acc=GSE18113) | GSM452738-40 | human lung microvascular endothelial cells |  |
| HSPCs | [GSE19429](http://www.ncbi.nlm.nih.gov/geo/query/acc.cgi?acc=GSE19429) | GSM483480-496 | MACS sorted CD34+ hematopoietic stem cells from bone marrow |  |
| HUVECs | [GSE16683](http://www.ncbi.nlm.nih.gov/geo/query/acc.cgi?acc=GSE16683) | GSM418126-28 GSM177134 GSM177140-42 | human umbilical vein endothelial cells |  |
| iPSCs | [GSE26672](http://www.ncbi.nlm.nih.gov/geo/query/acc.cgi?acc=GSE26672) | GSM656442-46 | induced pluripotent stem cells derived from fibroblasts |  |
| leukocytes | [GSE24598](http://www.ncbi.nlm.nih.gov/geo/query/acc.cgi?acc=GSE24598) | GSM250028-30 | MACS sorted CD45+ cells from bone marrow |  |
| macrophages | [GSE24598](http://www.ncbi.nlm.nih.gov/geo/query/acc.cgi?acc=GSE24598) | GSM250025-27 | MACS sorted CD11b+ cells from bone marrow |  |
| MAQC A | [GSE5350](http://www.ncbi.nlm.nih.gov/geo/query/acc.cgi?acc=GSE5350) | GSM122774-78 GSM122794-98 GSM122814-18 GSM122834-38 GSM122854-58 GSM122874-78 | brain tissue samples provided by microarray quality control consortium |  |
| MAQC B | [GSE5350](http://www.ncbi.nlm.nih.gov/geo/query/acc.cgi?acc=GSE5350) | GSM122779-83 GSM122799-803 GSM122819-23 GSM122839-43 GSM122859-63 GSM122879-83 | samples of 10 carcinoma cell lines provided by microarray quality control consortium |  |
| monocytes | [GSE16836](http://www.ncbi.nlm.nih.gov/geo/query/acc.cgi?acc=GSE16836) | GSM422109-16 | MACS sorted monocytes from peripheral blood |  |
| MSCs | [GSE12274](http://www.ncbi.nlm.nih.gov/geo/query/acc.cgi?acc=GSE12274) | GSM242185 GSM242673 GSM308224-28 | primary mesenchymal stromal cells from bone marrow |  |
| neutrophils | [GSE12662](http://www.ncbi.nlm.nih.gov/geo/query/acc.cgi?acc=GSE12662) | GSM317737-38 GSM317796 GSM317799-800 | FACS sorted CD9-CD14-CD15hiCD16hi neutrophil cells from BM |  |
| NSCs | [GSE15209](http://www.ncbi.nlm.nih.gov/geo/query/acc.cgi?acc=GSE15209) | GSM379865-69 | primary neuronal stem cells from fetal brain expanded in vitro |  |
| platelets | [GSE11524](http://www.ncbi.nlm.nih.gov/geo/query/acc.cgi?acc=GSE11524) | GSM290414-25 | preparation from platelet rich plasma from PB |  |
| promyelocytes | [GSE12662](http://www.ncbi.nlm.nih.gov/geo/query/acc.cgi?acc=GSE12662) | GSM317795 GSM317797-98 GSM317935 GSM318044 | FACS sorted CD9-CD14-CD15hiCD16lo promyelocytes from BM |  |
| spermatogonial cells | [GSE11350](http://www.ncbi.nlm.nih.gov/geo/query/acc.cgi?acc=GSE11350) | GSM282008 GSM282012-13 | MACS sorted CD49f+ spermatogonial cells from testis |  |
| T-cells | [GSE5788](http://www.ncbi.nlm.nih.gov/geo/query/acc.cgi?acc=GSE5788) | GSM135270-77 | MACS sorted CD3+ T-lymphocytes from peripheral blood |  |
| skin | GSE14905 | GSM372286-306 | Skin biopsies from healthy individuals |  |
| lung | GSE16538 | GSM415386-91 | Lung biopsies from healthy individuals |  |
| liver | GSE23343 | GSM572800-06 | Liver biopsies from healthy individuals |  |
| brain | GSE7621 | GSM184354-62 | Brain biopsies post-mortem; PMI = 9 h |  |
| skeletal muscle | GSE13205 | GSM333449-56 | Muscle biopsies from healthy individuals |  |
| heart | GSE1145 | GSM18442-52 | Left ventricle biopsies from healthy individuals | n/a |
| CD34-VSEL | GSE31869 | GSM790415 GSM790417 GSM790419 | FACS sorted CD34-VSEL cells from hUCB | this study |
| CD34+VSEL | GSE31869 | GSM790416 GSM790418 GSM790420 | FACS sorted CD34+VSEL cells from hUCB | this study |

Supplementary references

1. Gutierrez NC, Ocio EM, de Las Rivas J, Maiso P, Delgado M, et al. (2007) Gene expression profiling of B lymphocytes and plasma cells from Waldenstrom's macroglobulinemia: comparison with expression patterns of the same cell counterparts from chronic lymphocytic leukemia, multiple myeloma and normal individuals. Leukemia 21: 541-549.

2. Delorme B, Nivet E, Gaillard J, Haupl T, Ringe J, et al. (2010) The human nose harbors a niche of olfactory ectomesenchymal stem cells displaying neurogenic and osteogenic properties. Stem Cells Dev 19: 853-866.

3. Guenther MG, Frampton GM, Soldner F, Hockemeyer D, Mitalipova M, et al. (2010) Chromatin structure and gene expression programs of human embryonic and induced pluripotent stem cells. Cell Stem Cell 7: 249-257.

4. Laubli H, Spanaus KS, Borsig L (2009) Selectin-mediated activation of endothelial cells induces expression of CCL5 and promotes metastasis through recruitment of monocytes. Blood 114: 4583-4591.

5. Pellagatti A, Cazzola M, Giagounidis A, Perry J, Malcovati L, et al. (2010) Deregulated gene expression pathways in myelodysplastic syndrome hematopoietic stem cells. Leukemia 24: 756-764.

6. Sobrino A, Mata M, Laguna-Fernandez A, Novella S, Oviedo PJ, et al. (2009) Estradiol stimulates vasodilatory and metabolic pathways in cultured human endothelial cells. PLoS One 4: e8242.

7. Hu K, Yu J, Suknuntha K, Tian S, Montgomery K, et al. (2011) Efficient generation of transgene-free induced pluripotent stem cells from normal and neoplastic bone marrow and cord blood mononuclear cells. Blood 117: e109-119.

8. Shi L, Reid LH, Jones WD, Shippy R, Warrington JA, et al. (2006) The MicroArray Quality Control (MAQC) project shows inter- and intraplatform reproducibility of gene expression measurements. Nat Biotechnol 24: 1151-1161.

9. Wen Z, Wang C, Shi Q, Huang Y, Su Z, et al. (2010) Evaluation of gene expression data generated from expired Affymetrix GeneChip(R) microarrays using MAQC reference RNA samples. BMC Bioinformatics 11 Suppl 6: S10.

10. Ancuta P, Liu KY, Misra V, Wacleche VS, Gosselin A, et al. (2009) Transcriptional profiling reveals developmental relationship and distinct biological functions of CD16+ and CD16- monocyte subsets. BMC Genomics 10: 403.

11. Wagner W, Bork S, Horn P, Krunic D, Walenda T, et al. (2009) Aging and replicative senescence have related effects on human stem and progenitor cells. PLoS One 4: e5846.

12. Payton JE, Grieselhuber NR, Chang LW, Murakami M, Geiss GK, et al. (2009) High throughput digital quantification of mRNA abundance in primary human acute myeloid leukemia samples. J Clin Invest 119: 1714-1726.

13. Pollard SM, Yoshikawa K, Clarke ID, Danovi D, Stricker S, et al. (2009) Glioma stem cell lines expanded in adherent culture have tumor-specific phenotypes and are suitable for chemical and genetic screens. Cell Stem Cell 4: 568-580.

14. Raghavachari N, Xu X, Harris A, Villagra J, Logun C, et al. (2007) Amplified expression profiling of platelet transcriptome reveals changes in arginine metabolic pathways in patients with sickle cell disease. Circulation 115: 1551-1562.

15. Conrad S, Renninger M, Hennenlotter J, Wiesner T, Just L, et al. (2008) Generation of pluripotent stem cells from adult human testis. Nature 456: 344-349.

16. Durig J, Bug S, Klein-Hitpass L, Boes T, Jons T, et al. (2007) Combined single nucleotide polymorphism-based genomic mapping and global gene expression profiling identifies novel chromosomal imbalances, mechanisms and candidate genes important in the pathogenesis of T-cell prolymphocytic leukemia with inv(14)(q11q32). Leukemia 21: 2153-2163.

17. Yao Y, Richman L, Morehouse C, de los Reyes M, Higgs BW, et al. (2008) Type I interferon: potential therapeutic target for psoriasis? PLoS One 3: e2737.

18. Crouser ED, Culver DA, Knox KS, Julian MW, Shao G, et al. (2009) Gene expression profiling identifies MMP-12 and ADAMDEC1 as potential pathogenic mediators of pulmonary sarcoidosis. Am J Respir Crit Care Med 179: 929-938.

19. Misu H, Takamura T, Takayama H, Hayashi H, Matsuzawa-Nagata N, et al. (2010) A liver-derived secretory protein, selenoprotein P, causes insulin resistance. Cell Metab 12: 483-495.

20. Lesnick TG, Papapetropoulos S, Mash DC, Ffrench-Mullen J, Shehadeh L, et al. (2007) A genomic pathway approach to a complex disease: axon guidance and Parkinson disease. PLoS Genet 3: e98.

21. Fredriksson K, Tjader I, Keller P, Petrovic N, Ahlman B, et al. (2008) Dysregulation of mitochondrial dynamics and the muscle transcriptome in ICU patients suffering from sepsis induced multiple organ failure. PLoS One 3: e3686.
